# A LiF-Pie-Structured Interphase for Silicon Anodes

**DOI:** 10.1007/s40820-025-01832-y

**Published:** 2025-07-07

**Authors:** Weiping Li, Shiwei Xu, Cong Zhong, Qiu Fang, Suting Weng, Yinzi Ma, Bo Wang, Yejing Li, Zhaoxiang Wang, Xuefeng Wang

**Affiliations:** 1 Beijing National Laboratory for Condensed Matter Physics, Institute of Physics, Chinese Academy of Sciences, Beijing, 100190 People’s Republic of China; 2 College of Materials Science and Opto-Electronic Technology, University of Chinese Academy of Sciences, Beijing, 100049 People’s Republic of China; 3 State Key Laboratory of Space Power-Sources, School of Chemistry and Chemical Engineering, Harbin Institute of Technology, Harbin, 150001 People’s Republic of China; 4 Department of Energy Storage Science and Engineering, School of Metallurgical and Ecological Engineering, University of Science and Technology Beijing, Beijing, 100083 People’s Republic of China

**Keywords:** Si anodes, Solid electrolyte interface, Electrolyte additive

## Abstract

Silicon (Si) is a promising anode material for rechargeable batteries due to its high theoretical capacity and abundance, but its practical application is hindered by the continuous growth of porous solid-electrolyte interphase (SEI), leading to capacity fade. Herein, a LiF-Pie structured SEI is proposed, with LiF nanodomains encapsulated in the inner layer of the organic cross-linking silane matrix. A series of advanced techniques such as cryogenic electron microscopy, time-of-flight secondary ion mass spectrometry, and matrix-assisted laser desorption/ionization time-of-flight mass spectrometry have provided detailed insights into the formation mechanism, nanostructure, and chemical composition of the interface. With such SEI, the capacity retention of LiCoO_2_||Si is significantly improved from 49.6% to 88.9% after 300 cycles at 100 mA g^−1^. These findings provide a desirable interfacial design principle with enhanced (electro) chemical and mechanical stability, which are crucial for sustaining Si anode functionality, thereby significantly advancing the reliability and practical application of Si-based anodes.

**Supplementary Information:**

The online version contains supplementary material available at 10.1007/s40820-025-01832-y.

## Article Highlights

- A novel hierarchical solid electrolyte interface (SEI) structure is developed, featuring a lithium fluoride (LiF)-rich inner layer and a silane-based cross-linked matrix.
- A comprehensive suite of advanced characterization techniques provides multi-scale insights into the structural and chemical composition of the LiF-Pie SEI.
- The proposed SEI design significantly enhances cycling stability, achieving a capacity retention of LiCoO_2_||Si increase from 49.6% to 88.9% after 300 cycles at a current density of 100 mA g^−1^.

**Supplementary Information:**

The online version contains supplementary material available at 10.1007/s40820-025-01832-y.

## Graphical abstract

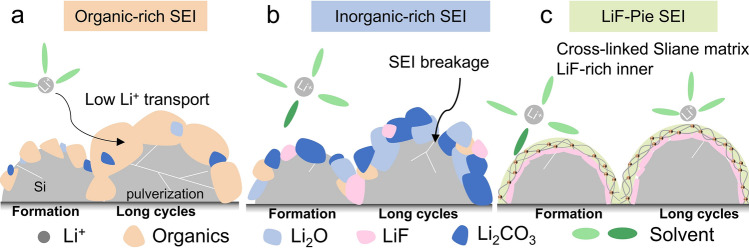

## Introduction

The development of high-performance lithium-ion batteries (LIBs) is crucial for advancing technologies such as electric vehicles, portable electronics, and grid energy storage systems, while ensuring high environmental sustainability and low cost [1–4]. Silicon (Si) has garnered much attention as a promising anode material for LIBs due to its exceptionally high theoretical capacity of approximately 3580 mAh g^–1^, significantly higher than that of conventional graphite anodes [5–8]. However, the practical application of Si anode is largely hindered by the instability of the solid-electrolyte interphase (SEI) during cycling which leads to mechanical degradation, loss of electrical contact, and ultimately reduces the battery’s performance and lifespan [9–11]. Therefore, it is imperative to design and develop a SEI with the desired properties to effectively protect Si-based anodes.

**Scheme 1:**
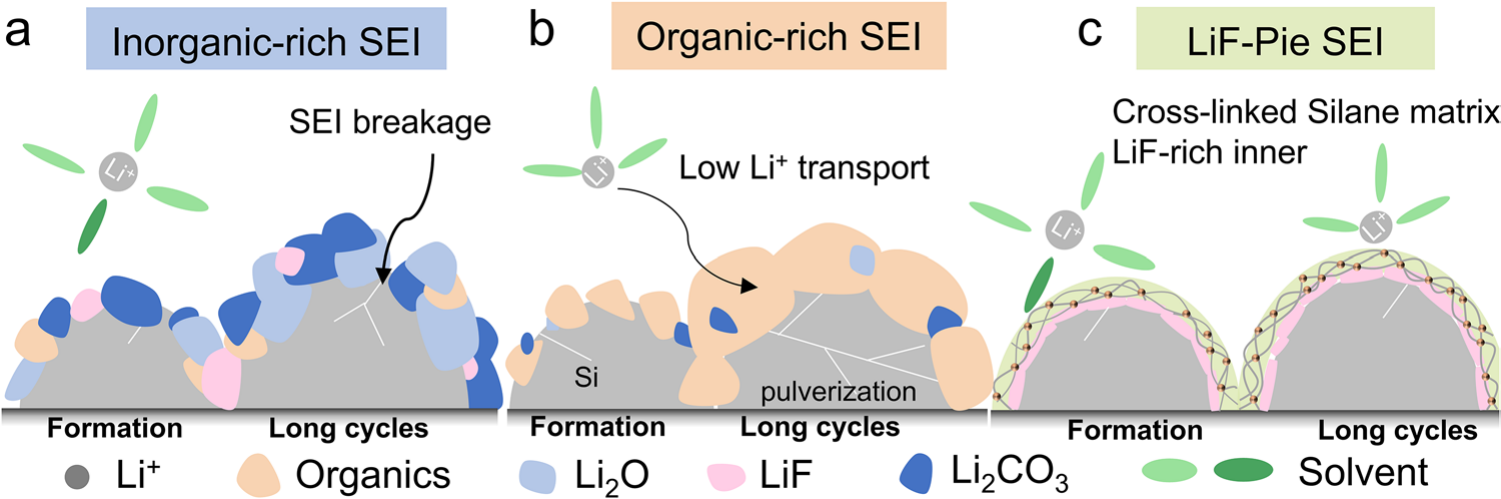
**a** Schematic illustration for the organic-rich SEI. **b** Schematic illustration for the inorganic-rich SEI. **c** Schematic illustration for the LiF-Pie structured SEI

**Fig. 1:**
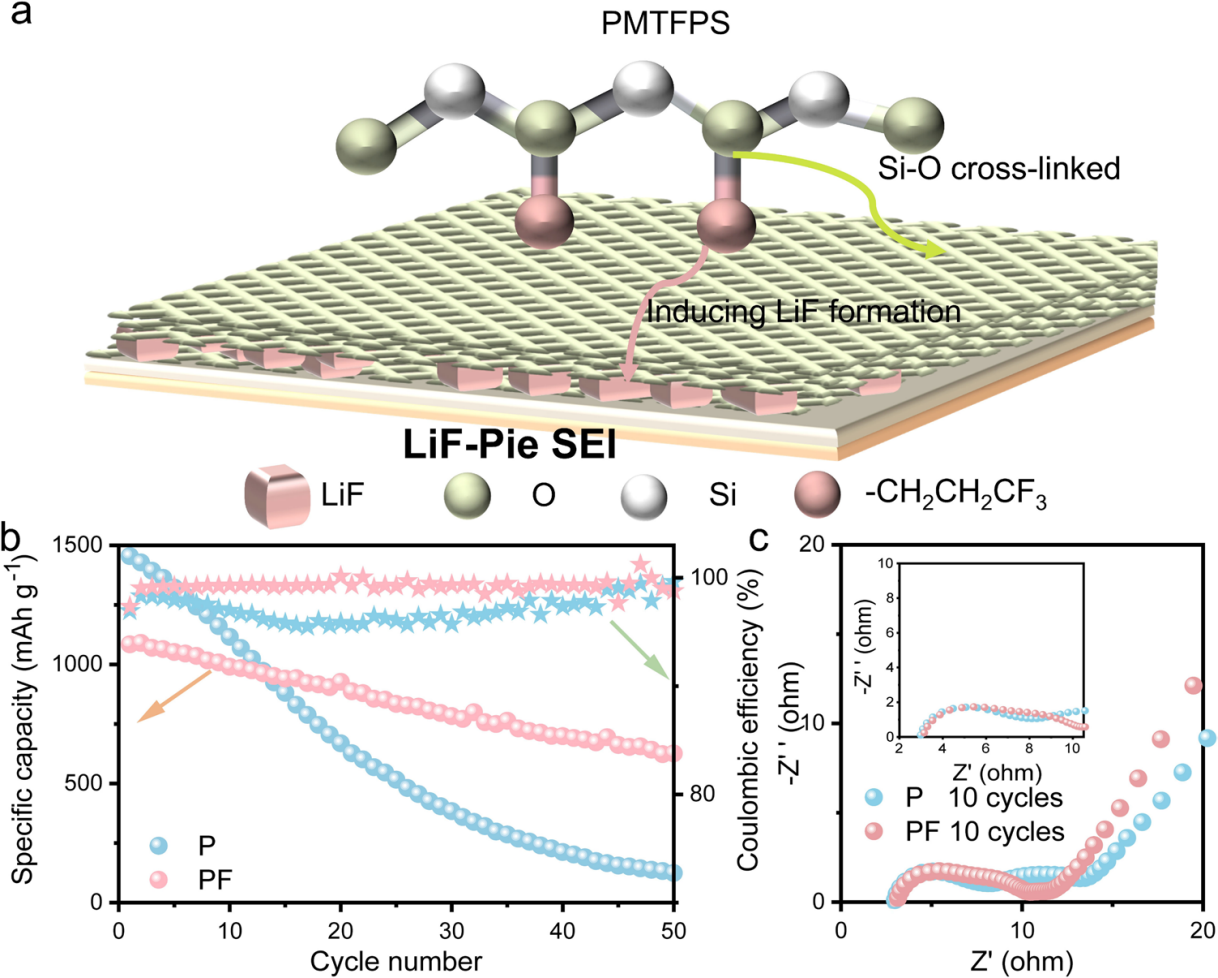
**a** Schematic diagram of the molecular structure and potential decomposition pathways of PMTFPS. **b** Long-term cycling performance of Si||Li cells with a voltage window of 0.005-3 V at 200 mA g^–1^. **c** EIS spectra of Si||Li cells after 10 cycles in the P and PF electrolyte

The SEI formed in traditional carbonate-based electrolytes is predominantly composed of low-elasticity organic materials (e.g., ROCOOLi) and a low amount of unevenly distributed inorganic components (Li_2_O, Li_2_CO_3_), exhibiting brittle fracture behavior under Si anodes’ anisotropic expansion. To resolve this problem, the inorganic-rich SEI (Scheme 1a) developed through using concentrated electrolyte salts [12–14], fluorinated electrolytes [15, 16] or inorganic salts-based additives [17–19] to enhance the modulus of the SEI and thermodynamic stability, effectively suppressing the expansion of Si-based anodes. However, their intrinsic rigidity causes interfacial debonding, particularly for high-mass-loading Si anodes (> 3 mg cm^–2^), leading to rapid capacity fade. In recent years, researchers have explored the introduction of polymers and flexible materials to create SEIs that possess elasticity or self-healing properties to address the mechanical instability (Scheme 1b) [20–23]. These advanced SEIs can effectively mitigate stress accumulation on the Si surface during volume expansion and contraction, thus maintaining the integrity and stability of the SEI during cycles. However, these SEIs usually have low ionic conductivity due to blocked Li^+^ pathways, which in turn reduces its efficiency. As discussed above, the stability of the SEI depends primarily on dynamic thermodynamic stability, mechanical stability and good adhesion to Si surface. To address these requirements, we propose a novel LiF-Pie-structured SEI in this study (Scheme 1c). The term ^“^LiF-Pie^”^ derived from a popular food ^“^Apple Pie^”^ metaphorically describes the hierarchical structure of SEI: an inner layer rich in lithium fluoride (LiF) acts as the ^“^filling,^”^ providing high mechanical rigidity and thermodynamic stability to suppress electrolyte corrosion, while a cross-linked silane outer matrix serves as the ^“^crust,^”^ ensuring elasticity to accommodate Si volume changes and maintain SEI integrity. This dual-layer design synergistically enhances both mechanical and electrochemical stability (Fig. 1a). Notably, LiF is a stable inorganic component during cycling while Li_2_O can react with Si, yielding Li_2_SiO_3_ [24]. While previous LiF-enriched SEIs succeeded in elevating LiF content [16], the design of organic component engineering in maintaining interfacial coherence was overlooked. Silane is preferred since the containing Si–O bond exhibits partial double-bond characteristics and a high degree of ionicity (40%–50%), enhancing resilience that can more effectively accommodate volume changes, compared to the typical C–O bonds in conventional SEI [25–27]. Additionally, both the high bonding energy of Si–O and the high surface energy of LiF ensure the intact adhesion to the Si electrode [28, 29].

To achieve a LiF-Pie SEI, a novel silane-coupling-agent-like electrolyte additive, poly(methyl trifluoropropyl siloxane) (PMTFPS) was employed. Advanced characterization techniques such as cryogenic electron microscopy (cryo-EM), time-of-flight secondary ion mass spectrometry (ToF–SIMS), and matrix-assisted laser desorption/ionization time-of-flight mass spectrometry (MALDI-ToF–MS) were used to reveal the nanostructure and composition of the PMTFPS-derived SEI. The results show that the inner layer of the SEI is enriched with LiF, which ensures thermodynamic stability and facilitates ion transport while the organic crosslinking silane matrix is incorporated to guarantee the high elasticity and monolithic nature of the interface. With such-structured SEI, the capacity retention of LiCoO_2_(LCO)||Si is dramatically improved from 49.6% to 88.9% after 300 cycles at 100 mA g^−1^. This SEI design not only bolsters the SEI’s thermal and mechanical robustness but also preserves the interfacial elasticity and cohesion, which are paramount for enduring Si anode functionality, significantly advancing the reliability and practical application of Si-based anodes.

## Experimental Section

### Electrolyte Preparation

Poly(methyl trifluoropropyl siloxane) (PMTFPS, Thermo Scientific Chemicals, MW = 2400 Da) was added to the electrolyte and thoroughly stirred for 2 h before use. The whole process of electrolyte preparation was conducted in the Ar-filled glove box with water and oxygen concentration less than 0.1 ppm.

### Electrochemical Measurements

Electrochemical performances were evaluated in 2032-type coin cells. All the coin cells were fabricated in the Ar-filled glove box with water and oxygen concentration less than 0.1 ppm. To fabricate the Si electrodes, a slurry was first prepared by mixing Si (83 wt% Si nanograins imbedded into porous carbon, specific capacity − 1800 mAh g^−1^), polyacrylate composite binders (PAA, Aladdin, MW = 450,000 Da), and carbon black (super P, Timcal Ltd.) with the mass ratio of 8:1:1. In the following, the slurry was cast onto a copper foil, dried at 60 °C for 6 h, and further dried at 120 °C for 6 h in the vacuum oven. The typical mass loading of the Si anode is 2.5–3 mg cm^−2^.

In Si||Li coin cells, the prepared Si electrodes, Li foils (φ16.2 mm, 600 μm in thickness) and glass fiber (φ16.2 mm) were used as working electrodes, counter electrodes and separators, respectively. 180 μL electrolytes was employed in the coin cell.

Similarly, the slurry of LCO cathodes was prepared by mixing commercial LCO materials (Canrd New Energy Technology Co., Ltd.), poly(vinylidene fluoride) (PVDF, Canrd New Energy Technology Co., Ltd.) and super P (Timcal Ltd.) with the mass ratio of 8:1:1. The resultant slurry was cast on the Al foil and dried at 80 °C for 12 h in vacuum oven. The typical mass loading of the LCO cathode is 13–15 mg cm^−2^ and the corresponding areal capacity is 2–2.4 mAh cm^−2^.

As for the full cell, the as-prepared LCO electrode was utilized as cathode, and 120 μL electrolyte was used in one LCO||Si full cell. For long-term cycling, the Si anode was initially cycled for 10 cycles in Li||Si half-cells (200 mA g^–1^), simulating the formation process critical for Si anodes. For practical applications, the operation conditions such as temperature, current density, and working voltage should be optimized to achieve a stable SEI on the Si surface. The N/P ratio in this work ranges from approximately 1.1–1.4, calculated based on the areal capacities of the anode and cathode. The specific capacities, determined from Fig. S20 (anode: 1087 mAh g^−1^) and Fig. 5a (later) (cathode: 155 mAh g^−1^), yield the following areal capacities: Anode**:** 2.5–3.0 mg cm^−2^ ✗ 1087 mAh g^−1^ ✗ 0.785 cm^2^ (φ = 10 mm) ≈ 2.1–2.6 mAh; Cathode**:** 13–15 mg cm^−2^ ✗ 155 mAh g^−1^ ✗ 0.785 cm^2^ ≈ 1.6–1.8 mAh. All full-cell tests were conducted within a voltage window of 2.5–4.4 V at current densities ranging from 0.1 to 2 C (1 C = 200 mA g^−1^).

All the coin cells were tested on a NEWARE battery test system (CT-4008 T-5V6A-S1-F). EIS test of cells was conducted on an electrochemical workstation (BioLogic SP-200 system) under room temperature in the frequency range from 1 to 100 mHz with an AC signal of 10 mV.

### Instruments and Characterizations

Post-cycling electrode handling: After disassembly in an Ar-filled glove box (H_2_O/O_2_ < 0.1 ppm), Si electrodes were rinsed with dimethyl carbonate (DMC) to remove residual electrolyte salts.

Si electrodes sample after different cycles were milled through cross-section polisher (Fischione 1061 SEM Mill) under − 80 °C and transferred in a sealed holder from glove box to a scanning electron microscope (SEM, HITACHI Regulus 8100) for observation.

Fourier-transform infrared (FTIR) spectra were acquired under ATR mode with a diamond crystal on a Bruker ALPHA II instrument in an argon-filled glove box.

X-ray photoelectron spectroscopy (XPS) measurement was carried out on a Thermo Scientific ESCALAB 250 Xi instrument with monochromatic 150 W (Al Kα line) radiation. Electrode samples were loaded on a sealed holder and transferred from glove box to the vacuum chamber. The peak positions of spectra were calibrated using the C–C bond (284.8 eV) signal as reference.

Differential scanning calorimetry (DSC, NETZSCH STA 449 F3) measurements were carried out from 30 to 500 °C in a sealed aluminum pan at a rate of 10 °C min^−1^.

Cryo-(S)TEM characterizations were carried out using a JEOL JEM-F200 microscope under cryogenic temperatures (− 180 °C) at 200 kV. The powder sample for cryo-(S)TEM characterizations was scratched from Si electrode rinsed by DMC and dispersed on Cu grid. Then the grid was loaded on the cryo-TEM holder (Fischione 2550) equipped with a tip retraction device in the glove box and transferred into the JEOL JEM-F200 microscope without air exposure with the help of a sealing sleeve. Liquid nitrogen was added to the cryo-TEM holder and temperature of sample was ensured to stabilize at − 180 °C before observation under cryo-(S)TEM.

Matrix-assisted laser desorption/ionization time of flight mass spectrometry (MALDI-ToF–MS) measurement was performed by a Bruker UltrafleXtreme instrument.

The ions composition and distribution of the anode surface and depth sputtering area were characterized by Time-of-Flight secondary ion mass spectrometry (ToF–SIMS, ToF–SIMS 5 ION-ToF GmbH, Münster, Germany). ToF–SIMS was equipped with a 30 keV Bi_3_^+^ primary ion gun and a 2 keV Cs^+^ sputter gun, and an electron flood gun was used for charge neutralization. Electrodes were mounted on a sealed holder (ION-ToF sample plate) under Ar atmosphere and transferred to the ToF–SIMS chamber via a vacuum transfer module to prevent air exposure.

The Young’s modulus of the SEI on Si electrode was obtained by PeakForce QNM mode (Bruker Multimode 8) with the RTESPA-525 tip in an atomic force microscope. AFM measurements (PeakForce QNM mode) were conducted in an Ar-filled glove box (H_2_O/O_2_ < 0.1 ppm).

## Results and Discussion

### PMTFPS-Derived SEI

After screening various polymer molecules, PMTFPS was selected because it not only contains a dimethoxy silane segment but also features a fluorosubstituted carbon chain (Fig. 1a), which was expected to decompose electrochemically forming LiF and cross-linked silane at the same time on the Si particles. PMTFPS was added into the electrolyte (the resultant electrolyte was denoted as PF) and its concentrations was optimized to be 0.05 M (Fig. S1) based on the cycling performance of Si anode (− 83 wt% Si nanograins imbedded into porous carbon, Fig. S2) with an active material loading of 5.0–6.0 mAh cm^−2^, higher than the most reports of 1.0–3.8 mAh cm^−2^ [12, 16, 30–33]. Although addition of PMTFPS slightly decreases the initial Coulombic efficiency (ICE) of Si anode from 90.54% to 90.43% during the initial formation stage at 50 mA g^−1^ (Fig. S3), the CE in the subsequent cycles is increased from 98.73% to 99.19% when the current was increased to 200 mA g^−1^ (Fig. 1b). This indicates that the SEI formed in the PF electrolyte has better stability than that in the pristine electrolyte (denoted as P). Notably, the capacity retention is improved significantly, reaching 60% after 50 cycles compared to only 9% for the P electrolyte. The CE with the PF electrolyte maintains as high as 99.2%, indicating a marked suppression of parasitic reactions between the electrolyte and active material during cycling. In contrast, the CE with the P electrolyte is first decreased from 98.5% to 95.5% after 20 cycles and becomes unstable in the subsequent cycles (Fig. 1b). The slightly lower initial specific capacity with PMTFPS additive in Si||Li half-cells is due to the increased polarization at the Li metal counter electrode (Fig. S3). EIS measurement demonstrates that the resistance for the Li^+^ transport through the SEI is reduced from 3.40 to 2.35 Ω after 10 cycles with PMTFPS additive, and the charge transfer impedance is also decreased from 5.94 to 4.68 Ω (Fig. 1c), suggesting the formation of a SEI with enhanced ionic conductivity and stability.

### Composition of the PMTFPS-derived SEI

**Fig. 2:**
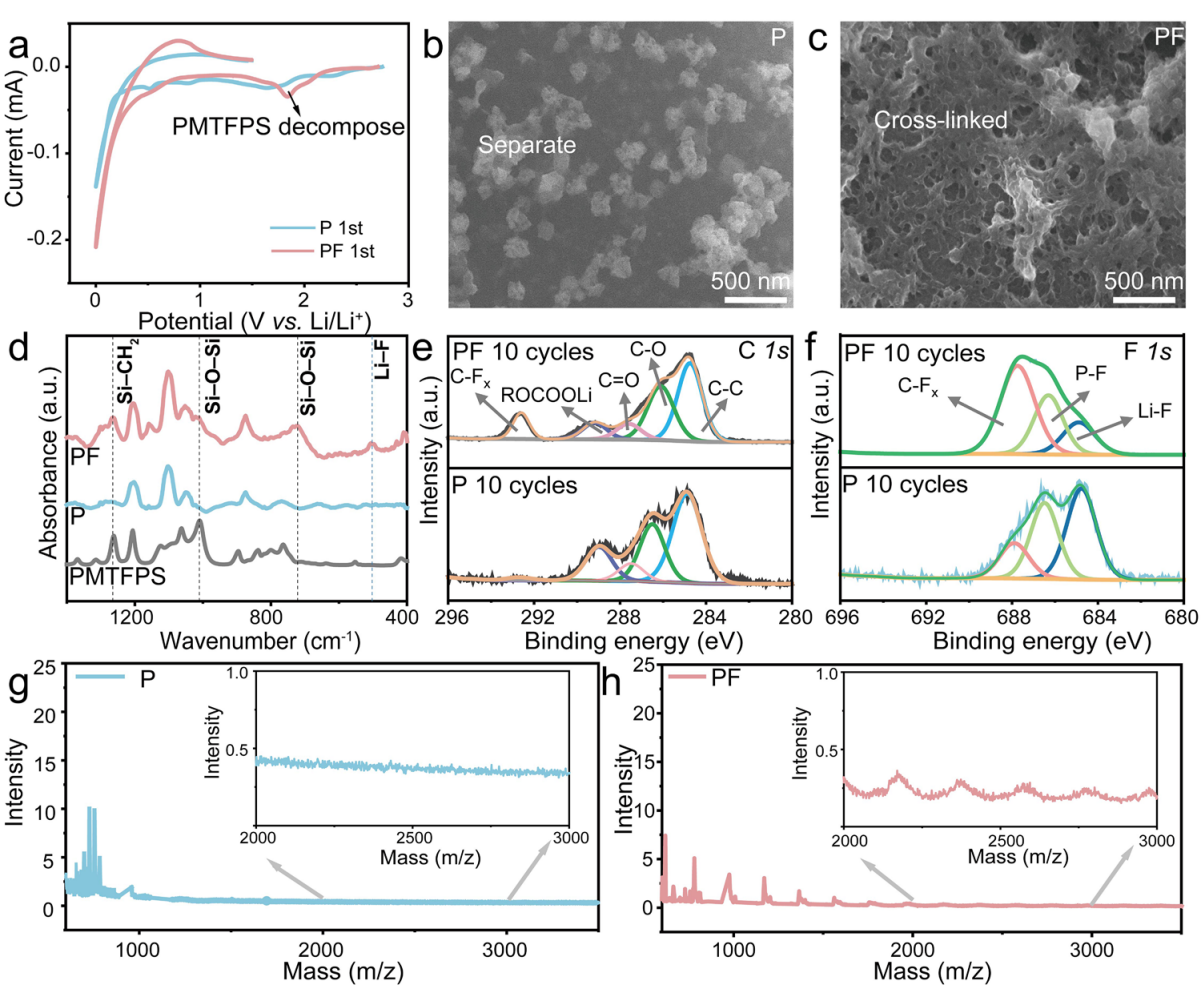
**a** Initial CV curves of Si anode. **b** Surface morphology images of Si particles with P electrolyte. **c** Surface morphology images of Si particles with PF electrolyte. **d** FTIR-ATR spectra of the Si anodes cycled in the P and PF electrolyte. **e** XPS spectra of the C *1 s* of the Si anodes cycled in the P and PF electrolyte. **f** XPS spectra of the F *1 s* of the Si anodes cycled in the P and PF electrolyte. **g** MALDI-ToF–MS spectra of the SEI formed in P electrolyte. **h** MALDI-ToF–MS spectra of the SEI formed in PF electrolyte

To understand the working principle of PMTFPS, cyclic voltammetry (CV) was first conducted, and a new reduction peak emerged around 1.8 V, corresponding to the electrochemical decomposition of PMTFPS (Fig. 2a). After a constant discharge at 1.8 V for 10 h, the SEI on individual Si particles cycled in the P electrolyte consists of isolated fragments, appearing as unevenly distributed nanoparticles (Figs. 2b and S4a). In contrast, the SEI on Si particles cycled in the PF electrolyte forms a continuous, cross-linked network, uniformly covering the particle surface (Figs. 2c and S4b). To elucidate the SEI’s evolution, top-view SEM images of Si anodes cycled in the PF electrolyte at different discharge voltages and holding times at 1.8 V were analyzed (Fig. S5). At 2.0 V, the SEI is almost invisible (Fig. S5a), indicating minimal formation. At 1.8 V, isolated SEI patches emerge (Fig. S5b), and at 1.6 V, the SEI begins to cover the particle surface (Fig. S5c). After holding at 1.8 V for 2 h (Fig. S5d), 5 h (Fig. S5e), and 10 h (Fig. S5f), the SEI evolves into a uniform, cross-linked network, indicative of a polymeric network. This continuous integrated SEI layer is advantageous in maintaining the interfacial integrity and minimizing SEI fracture against the volume changes, thus mitigating further electrolyte decomposition and consumption.

The chemical composition of the as-formed SEI was analyzed by attenuated total reflectance Fourier-transform infrared spectroscopy (ATR-FTIR, Fig. 2d), XPS (Fig. 2e, f), and MALDI-ToF–MS (Fig. 2g, h). The presence of characteristic molecular bands associated with PMTFPS in the ATR-FTIR spectra (Fig. 2d) suggests the formation of the PMTFPS-derived SEI. Additionally, more pronounced peaks corresponding to the symmetric stretching vibration of Si–O–Si bonds and Li–F bonds are present at the 1010, 721, and 504 cm^−1^, respectively, demonstrating the successful polymerization of silane incorporated with LiF [34, 35]. This is further confirmed by the higher concentration of elemental F (35.58 vs. 15.81 at%) and Si (7.08 vs. 0 at%) on the surface of the PMTFPS-derived SEI, along with the reduced concentrations of C and O (Fig. S6). Moreover, peaks belonging to Si–O (− 104.8 eV in Fig. S7) and C–F bonds (− 292.5 eV in Fig. 2e, and − 688.0 eV in Fig. 2f) are much enhanced in the XPS spectra of the PMTFPS-derived SEI. In contrast, less ROCOOLi (− 289.5 eV in Fig. 2e) and the P–F (− 686.3 eV in Fig. 2f) were found, suggesting that the preformed PMTFPS-derived SEI prevents the subsequent electrochemical decomposition of the pristine electrolyte [36, 37].

To further investigate the cross-linking polymeric species in the SEI, MALDI-ToF–MS was used due to its mild ionization capabilities, which preserves the integrity of polymeric chains and enables precise molecular weight analysis. This technique was critical for confirming the formation of a cross-linked polymer-dominated SEI in the PF electrolyte, contrasting with the small-molecule-rich SEI in the P electrolyte. The MALDI-ToF–MS spectrum of the original PMTFPS reveals a broad distribution with a dominant peak at 1400 Da and additional peaks extending up to − 3000 Da, reflecting its polydisperse nature (Fig. S8). The MALDI-ToF–MS spectrum of the SEI formed in the P electrolyte shows dominant peaks below 500 Da, indicating that its SEI mainly consists of small molecules (Fig. 2g) [38]. In contrast, the PF electrolyte SEI exhibits periodic peaks in the higher mass region (Da = 1200–3400, Fig. 2h), corresponding to siloxane oligomers with a degree of polymerization of 5–20 (based on a monomer unit mass of − 156 Da), consistent with the predominant retention of the original siloxane backbone of PMTFPS. These high-molecular-weight species (> 500 Da) confirm the presence of a cross-linked polymeric network, which enhances the SEI’s continuity and mechanical integrity, distinguishing it from the less cohesive SEI in the P electrolyte [39].

### LiF-Pie-Structured SEI

**Fig. 3:**
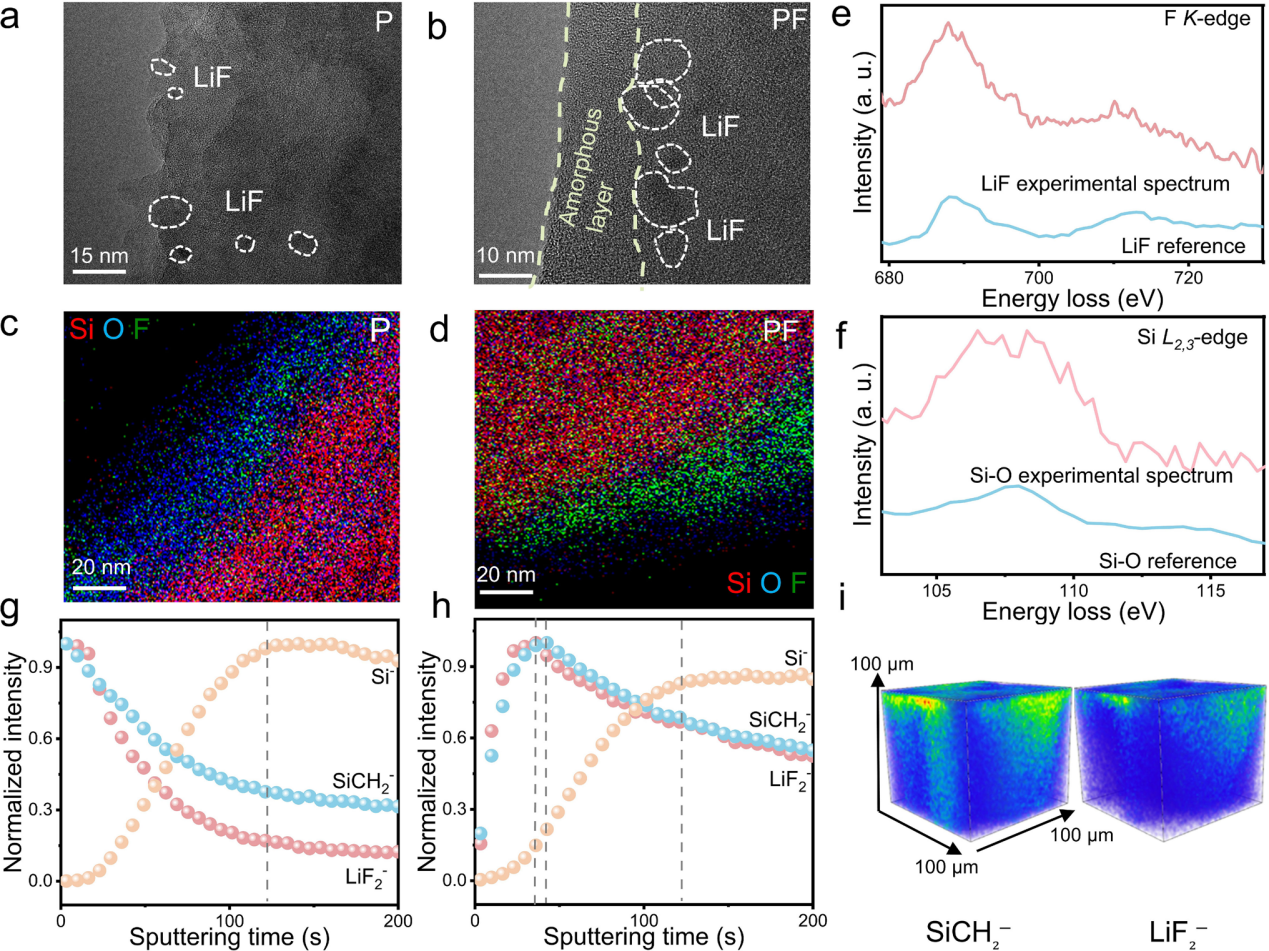
**a** Cryo-EM images of SEI formed in the P electrolyte.** b** Cryo-EM images of SEI formed in the PF electrolyte. **c** Corresponding distribution of the elemental O, Si and F based on the EDS mapping from the Si electrodes cycled in the P electrolyte. **d** Corresponding distribution of the elemental O, Si and F based on the EDS mapping from the Si electrodes cycled in the PF electrolyte. **e** EELS spectra of F *K*-edge with their references. **f** EELS spectra of Si *L*_*2,3*_-edge with their references **g** Depth profiles of Si^–^, LiF_2_^–^, and SiCH_2_^–^ from the SEI formed in the P electrolyte; **h** Depth profiles of Si^–^, LiF_2_^–^, and SiCH_2_^–^ from the SEI formed in the PF electrolyte. **i** ToF–SIMS secondary ion 3D images of the LiF_2_^−^ and SiCH_2_^−^ (scale 100 μm × 100 μm × 100 μm) on the Si anode cycled in the PF electrolyte

The nanostructure of the PMTFPS-derived SEI was probed by cryo-EM and ToF–SIMS. As shown in Fig. 3a, the SEI formed in the P electrolyte is dominated by amorphous organics with several crystalline LiF nanograins (enlarged images in Fig. S9) sporadically dispersed in it. In contrast, the PMTFPS-derived SEI displays a distinct two-layer structure with an inner layer rich in LiF (Figs. 3b and S10). Energy-dispersive spectroscopy (EDS) mappings provide additional confirmation, elucidating the distinct elemental distributions within the SEI layers formed in the P and PF electrolytes. The former exhibits random distribution of O and F on the surface of Si particle (Figs. 3c and S11) while the latter showcases a preferential F enrichment in the inner layer, forming a LiF-rich region close to the Si particle that is crucial for enhancing the mechanical strength of the SEI (Figs. 3d and S12). The presence of the LiF in the inner layer is further confirmed by EELS spectra (Fig. 3e) and its mapping (Fig. S13) without any detectable F signal on the outer layer (Fig. S14). Additionally, strong signals belonging to the Si–O bonding were detected in the Si *L*_2,3_-edge spectrum from the outer layer (Fig. 3f), indicating that it is mainly made of silane [40].

To gain more detailed insights into the elemental content and spatial distribution of the two SEI types at a large scale (micrometer), time-of-flight secondary ion mass spectrometry (ToF–SIMS) analysis was conducted. Since the Si^–^ signals from the Si bulk are stabilized more rapidly with the addition of PMTFPS (Figs. 3g, h and S15), its SEI is thinner than the pristine one (Fig. 3g). The content of LiF_2_^–^ and SiCH_2_^–^ is decreased sharply in the original electrode at the onset of sputtering (Figs. 3g and S15a), whereas they are centered at around 40-s sputtering time in the PF system (Figs. 3h and S15b), suggesting that the LiF is rich in the inner layer incorporated in the silane substrate. Such structure of PMTFPS-derived SEI is further confirmed by the three-dimensional distribution mappings of LiF_2_^–^ and SiCH_2_^–^ (Fig. 3i). Therefore, a LiF-Pie structured SEI derived from PMTFPS is clearly visualized by cryo-TEM and ToF–SIMS.

### Merits of the LiF-Pie-Structured SEI

**Fig. 4:**
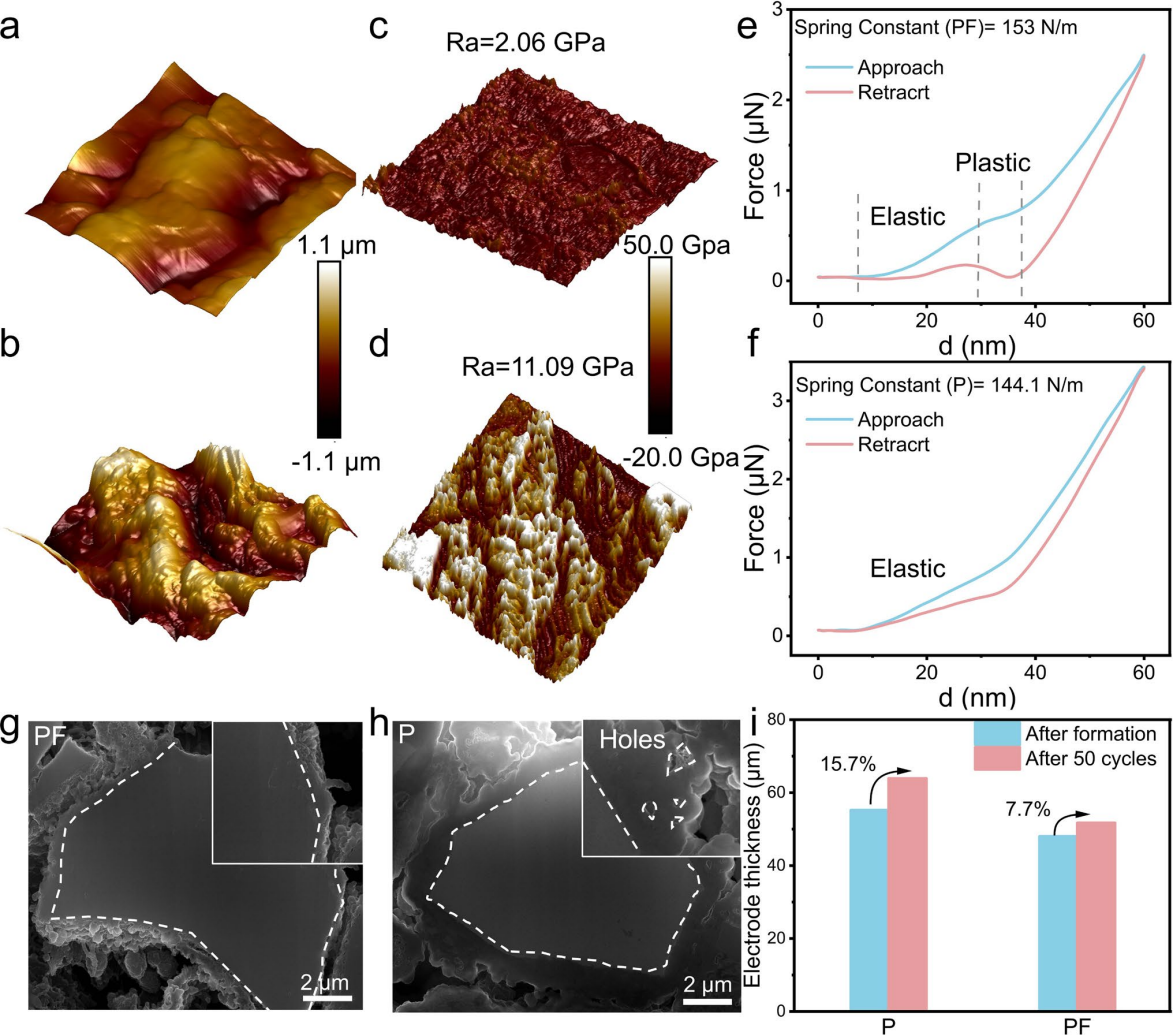
Typical AFM morphology (3 μm × 3 μm) of the SEI after 30 cycles in **a** PF electrolyte, **b** P electrolyte. Young’s modulus (3 μm × 3 μm) of the SEI after 30 cycles in **c** PF electrolyte, **d** P electrolyte. Force–displacement curves comparison of the SEI after 30 cycles in **e** PF electrolyte, **f** P electrolyte. Cross-sectional SEM images of the Si particles after 50 cycles in **g** PF electrolyte, **h** P electrolyte. **i** Electrode thickness changes of the Si anode before and after 50 cycles in the P and PF electrolytes at 200 mA g^−^.^1^

The LiF-Pie-structured SEI exhibits a smoother particle surface with reduced roughness, as evidenced by atomic force microscopy (AFM) measurements (Fig. 4a, b). Additionally, the top surface of the cross-linked SEI shows a significantly lower Derjaguin-Muller-Toporov (DMT) modulus (2.06 *vs.* 11.09 GPa, Fig. 4c, d), indicating enhanced tolerance to volume changes. This improved mechanical behavior is further supported by the higher average spring constant of the LiF-rich SEI (153 *vs.* 144.3 N m^−1^, Fig. 4e, f), derived from force–displacement curves. The increased spring constant underscores the superior flexibility and robustness of the LiF-rich SEI [38, 41]. The presence of the plastic LiF-rich inner layer is also beneficial for inhibiting the volume changes and maintaining the integrity of both surface and bulk (Fig. 4e). As a consequence, a thin (< 0.2 μm), dense, and uniform SEI layer (Figs. 4g and S15) is found on the Si particle after 50 cycles in the PF electrolyte while a thick (> 1.5 μm), and porous corrosive film rich in C, F, and O are present in that cycled in the P electrolyte (Figs. 4h and S16). Moreover, the swelling rate of the electrode thickness is much reduced from 15.7% to 7.7% (Figs. 4i and S18). Therefore, the LiF-Pie structured SEI is stable and contributes to suppressing the parasitic reactions between the electrolyte and Si as well as electrode expansion, which is critical for the practical application of Si-based LIBs. Additionally, it also exhibits higher thermal stability with a higher exothermic temperature (83.2 *vs.* 80.1 °C) and lower heat release (21.2 *vs.* 25.3 mW mg^–1^) as confirmed by the differential scanning calorimetry analysis (Fig. S19).

### Full Battery Test

**Fig. 5:**
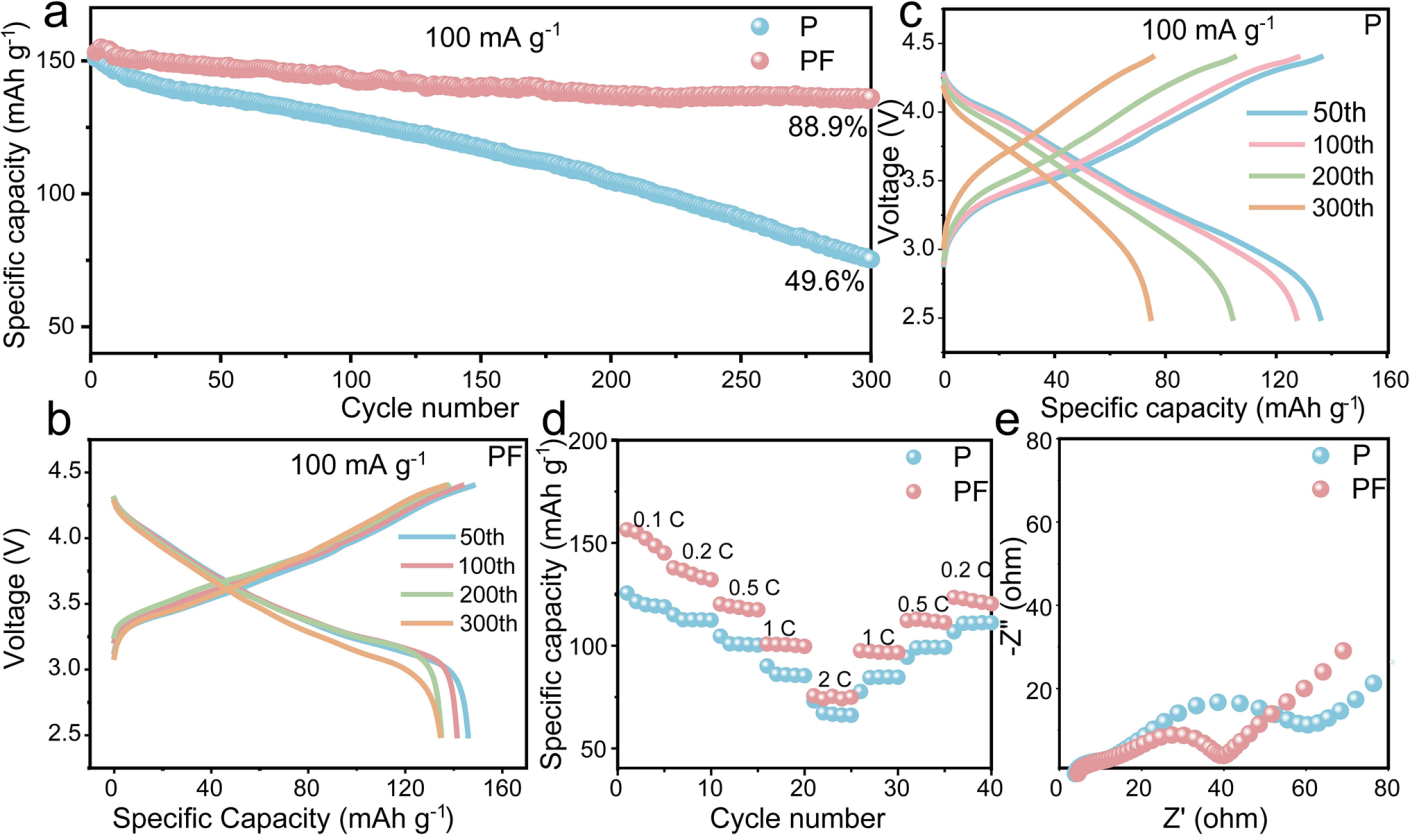
**a** Cycling performance of LCO||Si cells at 100 mA g^–1^. The charging/discharging profiles of the LCO||Si cells at some selected cycles in **b** PF electrolytes, **c** P electrolytes. **d** Rate performance. **e** EIS spectra of the LCO||Si cells after 10 cycles in the P and PF electrolytes

The benefit of the LiF-Pie SEI was further validated in the LCO||Si full cell with a N/P ratio of 1.1–1.4 (Fig. S20). As anticipated, the LCO||Si cell with PF electrolyte delivers a reversible capacity of 150 mA g^−1^ at a charge/discharge rate of 0.5 C and achieves remarkable capacity retention of approximately 88.9% after 300 cycles, demonstrating its superior electrochemical cycling stability (Fig. 5a). To further contextualize the performance of the LiF–Pie-structured SEI, we compared our results with recent advances in silicon anode stabilization strategies, as summarized in Table S2. As shown, the PMTFPS-derived SEI outperforms most previously reported approaches. Furthermore, the CE remains exceptionally high (> 99.35%) throughout the cycling process (Figs. 5b and S21), indicating the minimal side reactions prevented by the LiF-Pie SEI, which is crucial for maintaining the battery’s energy density and longevity. In contrast, the capacity of LCO||Si cell with P electrolyte is dropped fast and its capacity retention after 300 cycles is only 49.6% (Figs. 5c and S17). The corresponding average CE is less than 98.78%, indicating the occurrence of continuous side reactions. Since both LCO particles cycled in the P and PF electrolyte reveal a uniform 3–4 nm cathode electrolyte interphase (Fig. S22), the positive effect of PF additive is mainly on the Si anode.

The LCO||Si cell with the PF electrolyte exhibits superior rate performance than that with the P electrolyte across a range of rates from 0.1 to 2 C (Fig. 5d). For instance, the reversible capacity at 1 C is 100.6 *vs.* 86.1 mAh g^–1^. Further impedance tests (Fig. 5e) indicate that the charge transfer impedance in the PF electrolyte (19.92 Ω) is lower than that in the P electrolyte (44.45 Ω) after 10 cycles. To evaluate the long-term stability of the LiF–Pie SEI, top-view SEM images of Si anodes after 300 cycles were analyzed (Fig. S23). In the PF electrolyte, the Si surface remains covered by a uniform and intact LiF–Pie SEI, without noticeable cracking or particle pulverization (Fig. S23b). In contrast, the P electrolyte results in a rough, fractured SEI and visibly degraded Si particles (Fig. S23a). Complementary electrochemical impedance spectroscopy (EIS) measurements after 300 cycles (Fig. S24) further confirm the superior interfacial stability of the LiF–Pie SEI. The PF electrolyte exhibits a significantly lower charge transfer resistance (78.07 Ω) compared to the P electrolyte (104.7 Ω), indicating better preserved ionic transport and mechanical integrity over extended cycling. Therefore, the LiF-Pie SEI exhibits high stability and ionic conductivity, prolonging the cycling lifespan and facilitating the reaction kinetics of LIBs with high energy density.

## Discussions

As previously mentioned, the LiF-Pie-structured SEI derived from PMTFPS significantly enhances the electrochemical performance of Si anodes by forming a stable, conductive, and flexible SEI layer. This structure effectively mitigates volume expansion, suppresses parasitic reactions, and improves cycling stability, rate performance, and thermal stability, providing substantial advantages for high-energy–density LIBs. Additionally, the PF electrolyte exhibits superior fire resistance, with a notably shorter self-extinguishing time (SET, 10.0 vs. 67.5 s g^–1^, Figs. S25 and S26) and enhanced thermal stability (Fig. S27). The PF electrolyte also demonstrates improved cathodic stability, with a threshold voltage of 5.3 V, compared to 4.65 V for the P electrolyte (Fig. S28).

## Conclusion

In summary, the innovative use of PMTFPS as a multifunctional electrolyte additive has significantly stabilized Si anodes by in situ forming a LiF-Pie-structured SEI. This SEI consists of a flexible, cross-linking matrix and a LiF-rich inner layer, which collectively enhance the SEI’s flexibility, mechano-electrochemical properties, and thermal stability. Consequently, the PF electrolyte, incorporating this optimized SEI design, demonstrated substantial improvements in the CE, rate capability, and cycle stability compared to the P electrolyte for LCO||Si cells. This study highlights the critical role of SEI structure design in developing electrolytes that meets the high elasticity and stability demands of practical, thick Si anodes, offering valuable insights for future electrolyte formulations.

## Supplementary Information

Below is the link to the electronic supplementary material.

Supplementary file1 (DOCX 29675 KB)
