# Supplementary material for: A LiF-Pie-Structured Interphase for Silicon Anodes
Source: Nanomicro Lett. 2025 Jul 7;17:322. doi: 10.1007/s40820-025-01832-y (PMC12229977; doi:10.1007/s40820-025-01832-y)
Supplement: Supplementary file 1 — Supplementary file1 (DOCX 29675 KB) [file 40820_2025_1832_MOESM1_ESM.docx]

Supporting Information for

**A LiF-Pie Structured Interphase for Silicon Anodes**

Weiping Li^1,2^, Shiwei Xu^1^, Cong Zhong^1^, Qiu Fang^1,2^, Suting Weng^1^, Yinzi Ma^1^, Bo Wang^3^, Yejing Li^4^, Zhaoxiang Wang^1,2^, Xuefeng Wang^1,2*^

1 Beijing National Laboratory for Condensed Matter Physics, Institute of Physics, Chinese Academy of Sciences, Beijing 100190, P. R. China

2 College of Materials Science and Opto-Electronic Technology, University of Chinese Academy of Sciences, Beijing 100049, P. R. China

3 State Key Laboratory of Space Power-Sources, School of Chemistry and Chemical Engineering, Harbin Institute of Technology, Harbin 150001, P. R. China

4 Department of Energy Storage Science and Engineering, School of Metallurgical and Ecological Engineering, University of Science and Technology Beijing, Beijing 100083, P. R. China

*Corresponding author. E-mail: [wxf@iphy.ac.cn](mailto:wxf@iphy.ac.cn) (Xuefeng Wang)

**Supplementary Figures**


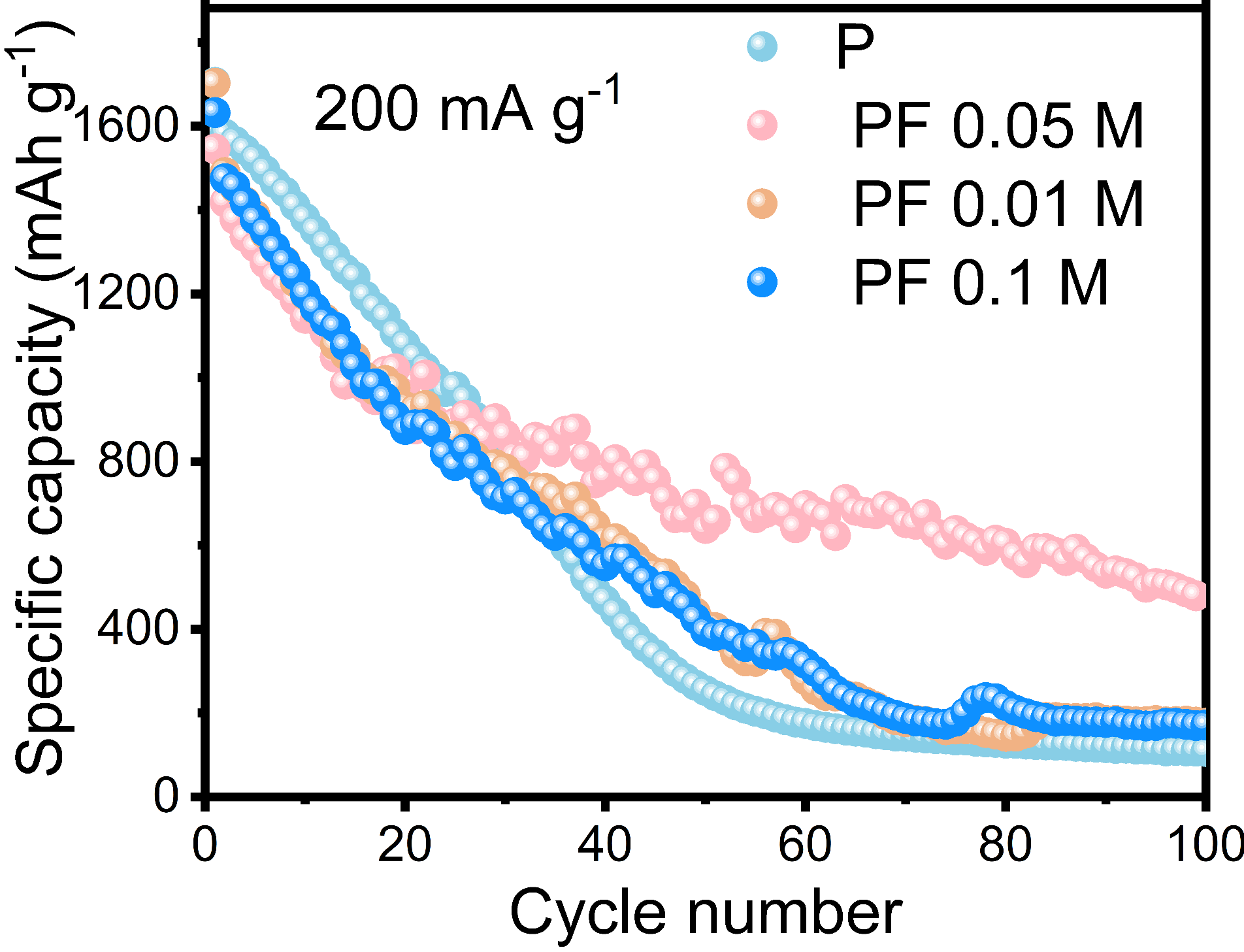


**Fig. S1** The cycle performance of Si anode with different concentrations of PMTFPS at current density of 200 mA g^−1^


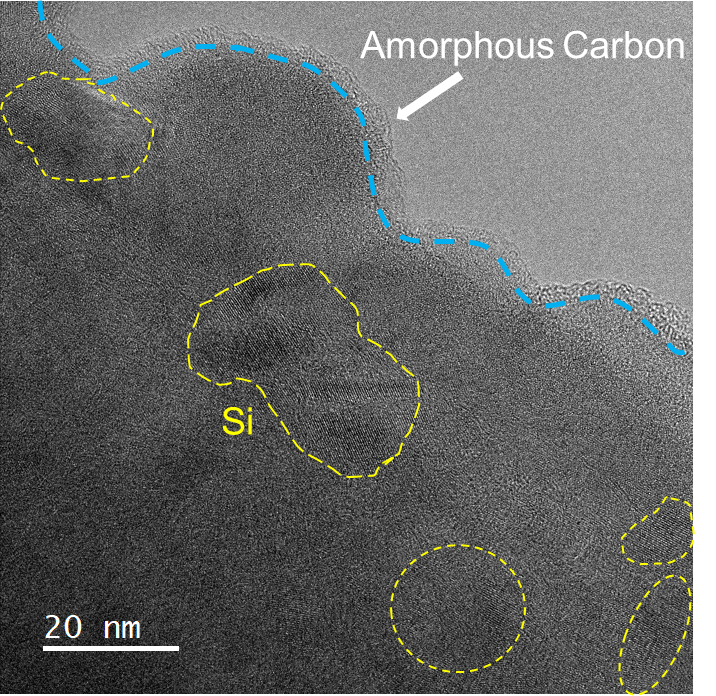


**Fig. S2** High-resolution transmission electron microscopy image of the pristine Si particle





**Fig. S3** (**a**) Initial charge/discharge profiles of Si anode at 50 mA g^–1^ for the first three cycles and 200 mA g^–1^ for the subsequent cycles; (**b**) Cyclic performances of Li−Li symmetric cells under 1.0 mA cm^−2^/1.0 mAh cm^−2^

**Fig. S4** Top-view SEM images of Si anodes cycled in the (**a**) P and (**b**) PF electrolyte
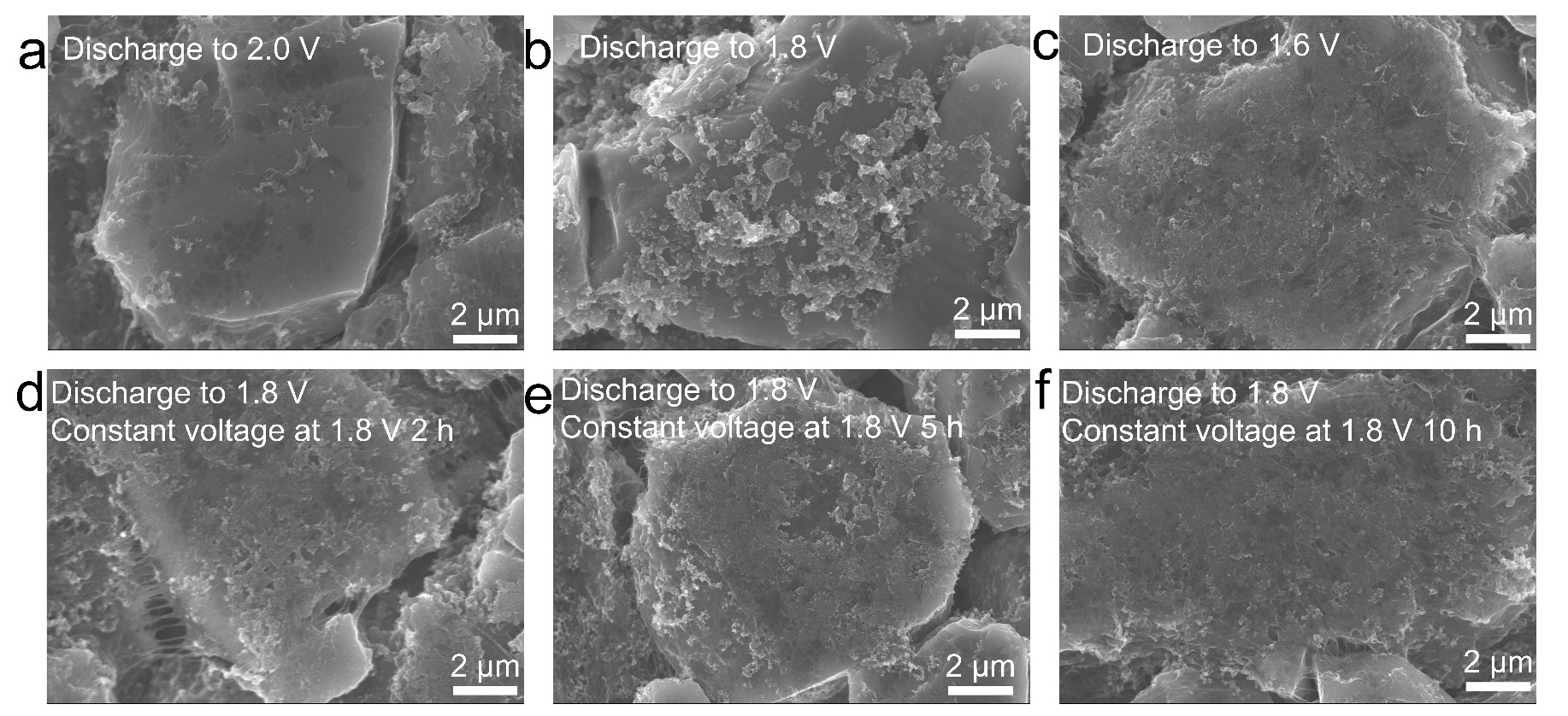


**Fig. S5** Top-view SEM images of Si anodes cycled in the PF electrolyte discharging to 2.0 V (**a**), 1.8 V (**b**) and 1.6 V (**c**); and applied a constant voltage of 1.8 V for 2 h (**d**), 5 h (**e**), 10 h (**f**)


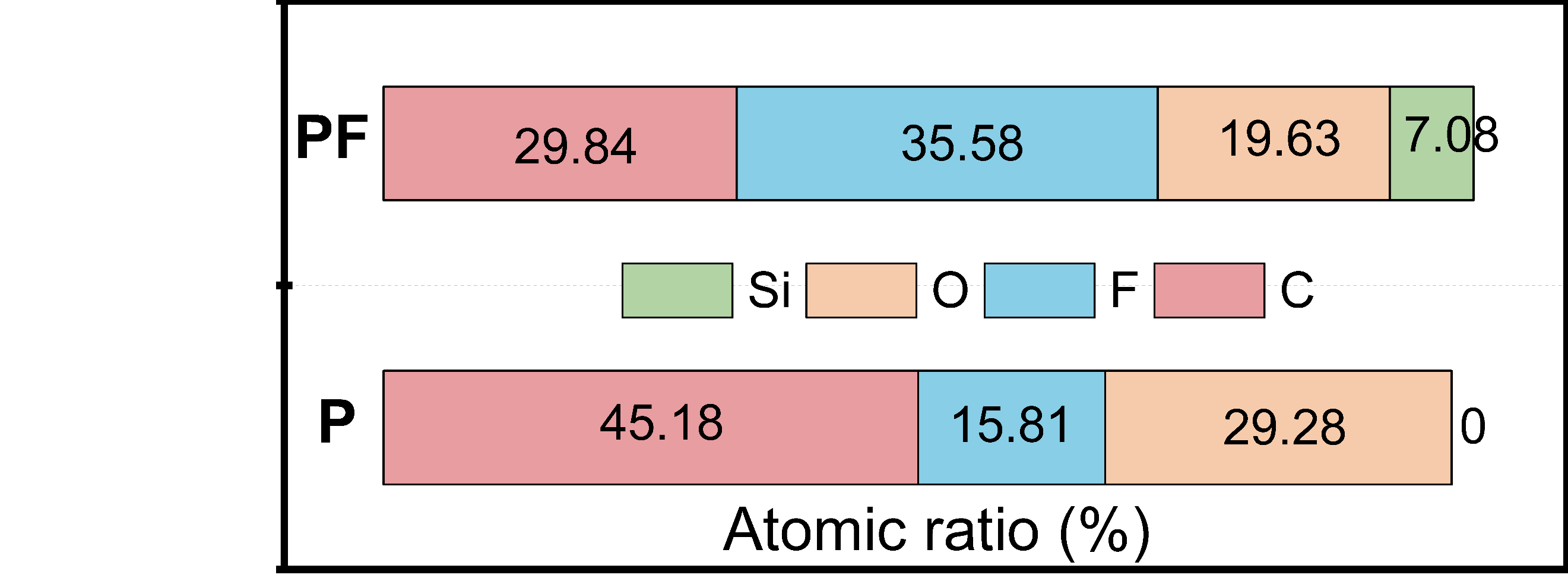


**Fig. S6** Relative atomic content of Si, O, F, and C based on the XPS survey spectra of the SEI formed in the P and PF electrolytes


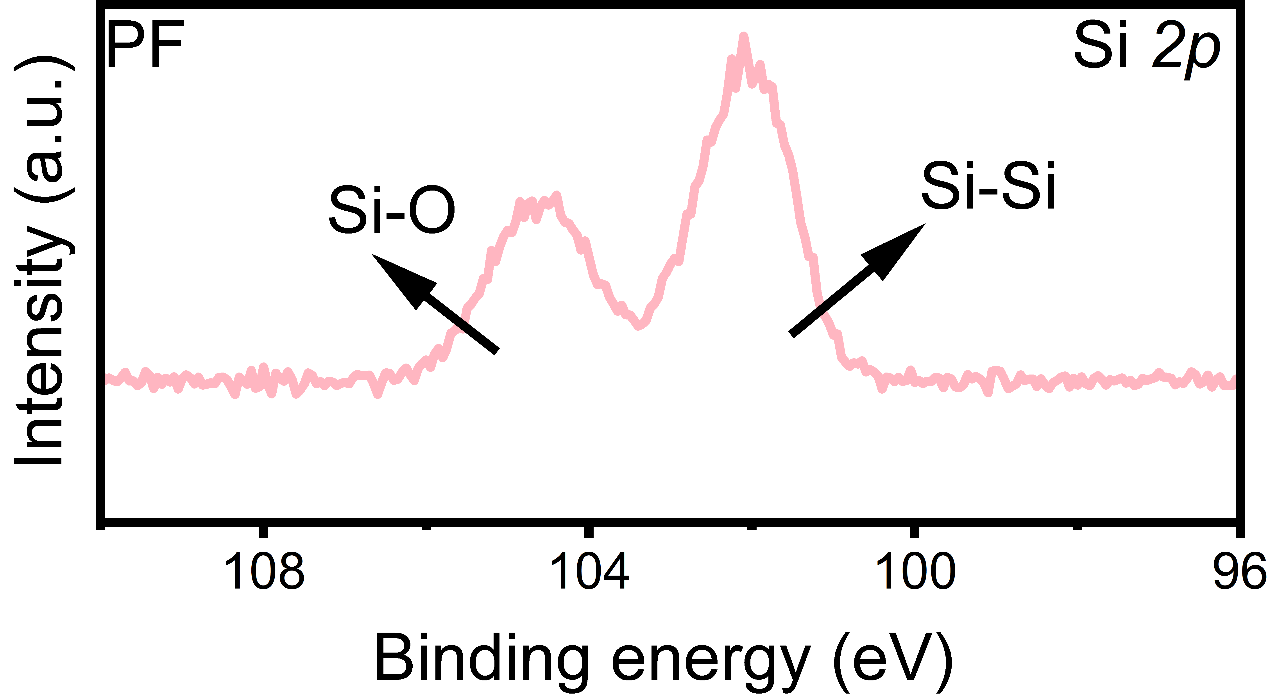


**Fig. S7** XPS spectrum of the Si *2p* from the Si anode cycled in the PF electrolyte


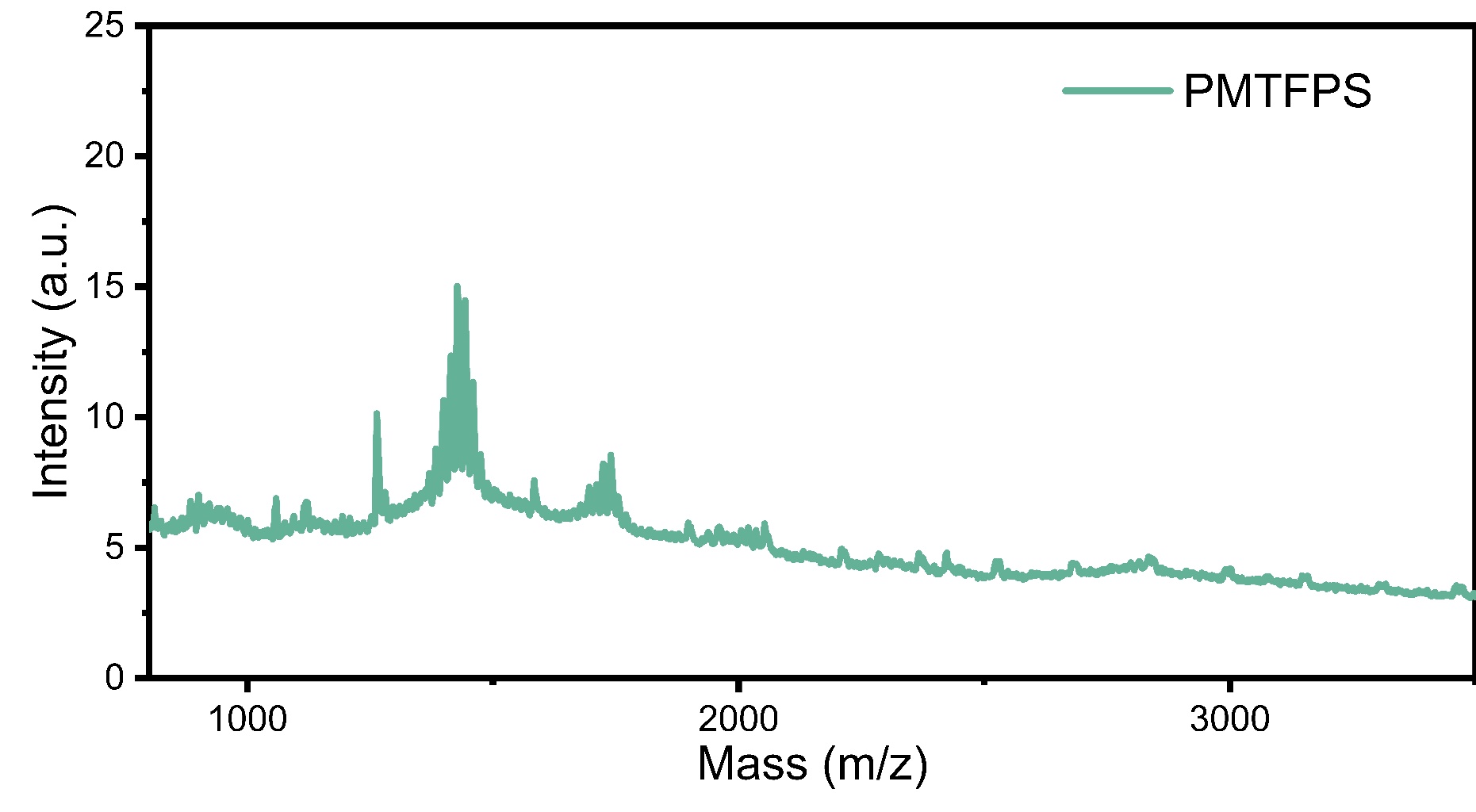


**Fig. S8** MALDI-ToF-Ms spectrum of PMTFPS


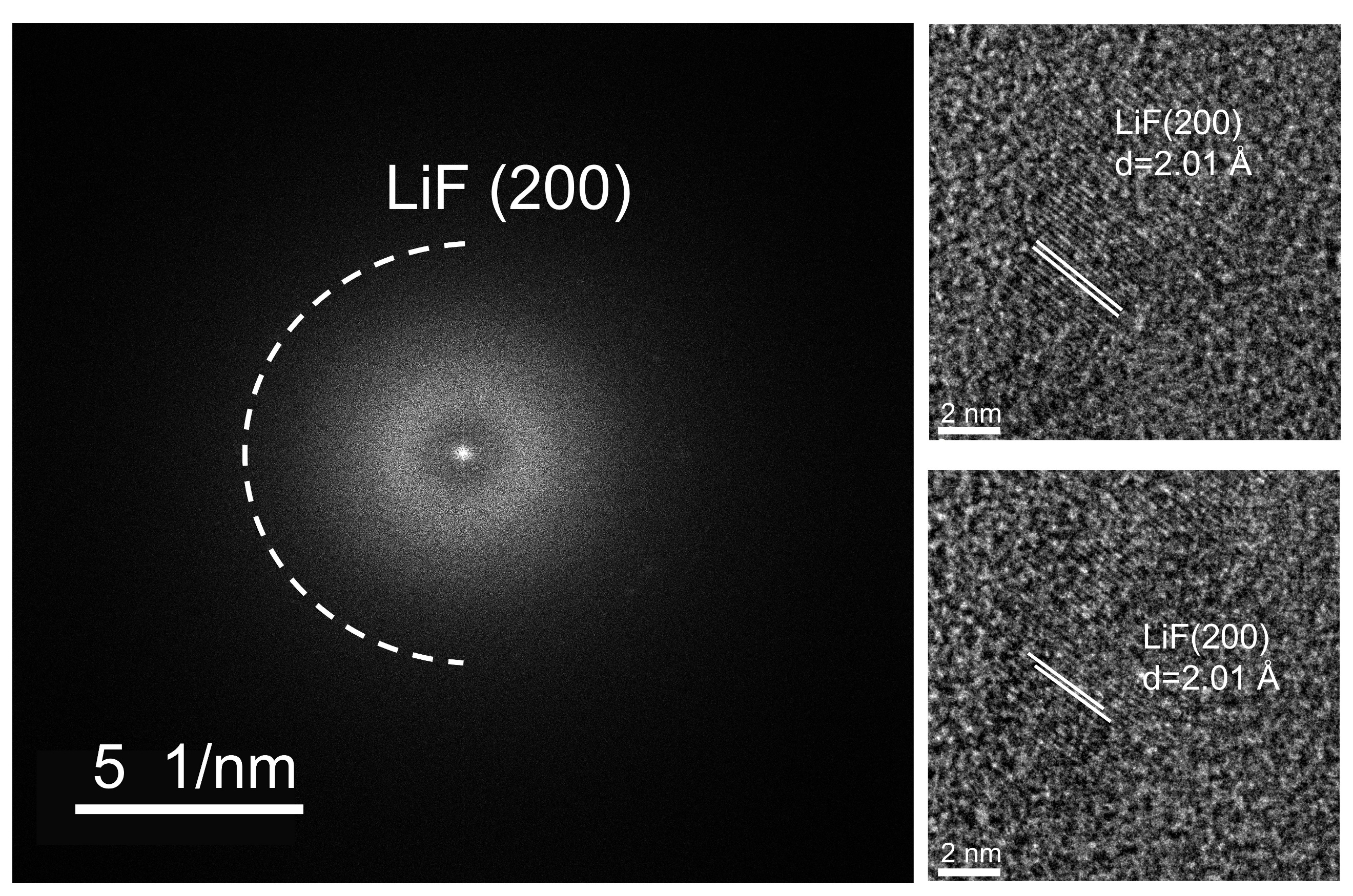


**Fig. S9** The corresponding FFT pattern of the Fig. 3a and the enlarged region of crystalline LiF


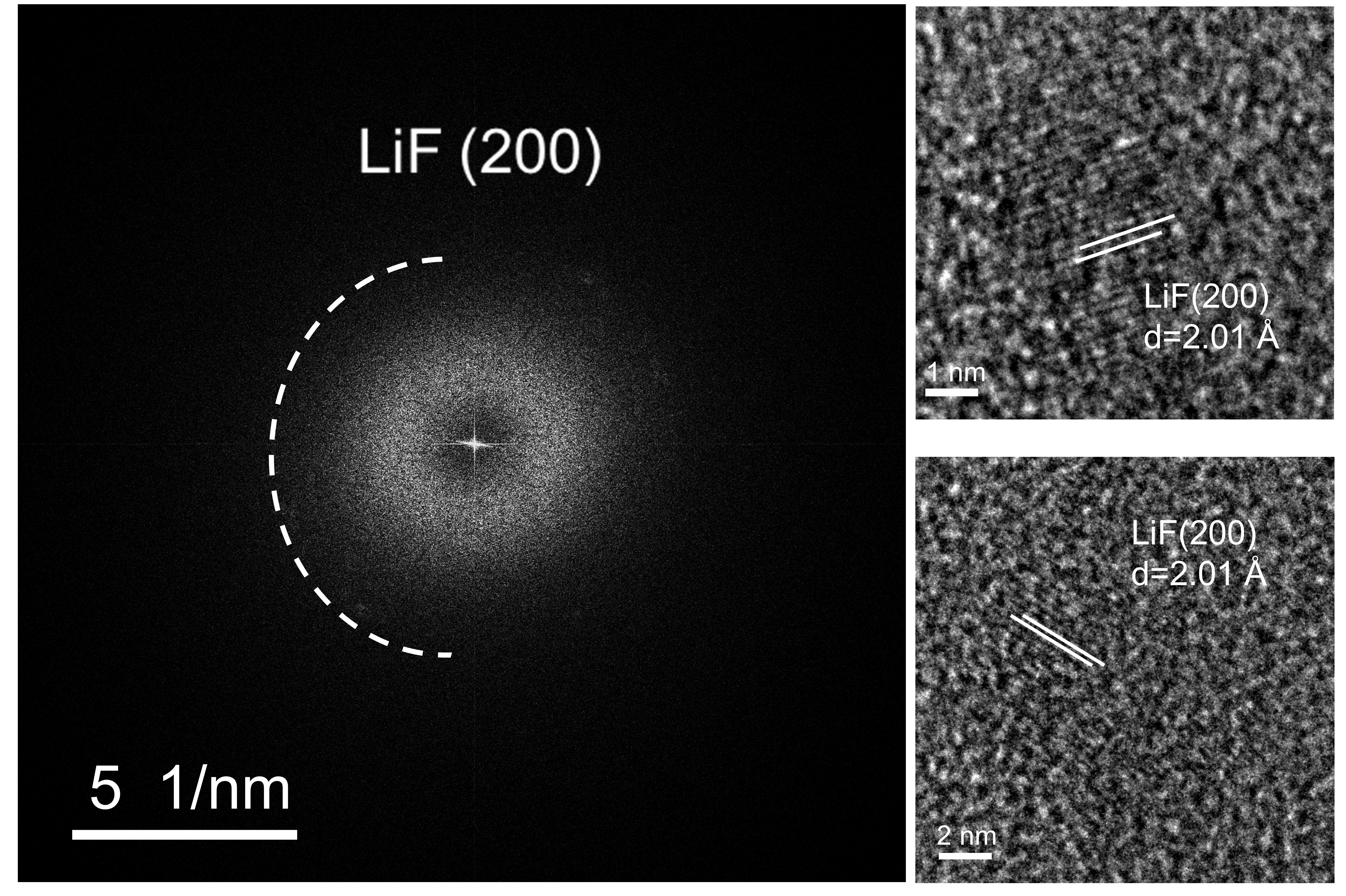


**Fig. S10** The corresponding FFT pattern of the Fig. 3b and the enlarged region of crystalline LiF


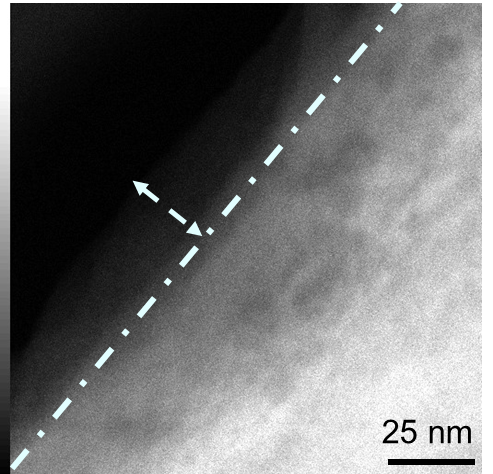


**Fig. S11** Dark-field scanning transmission electron microscopy (STEM) image of the Si anode cycled in P electrolyte for the EDS mapping in the Fig. 3c


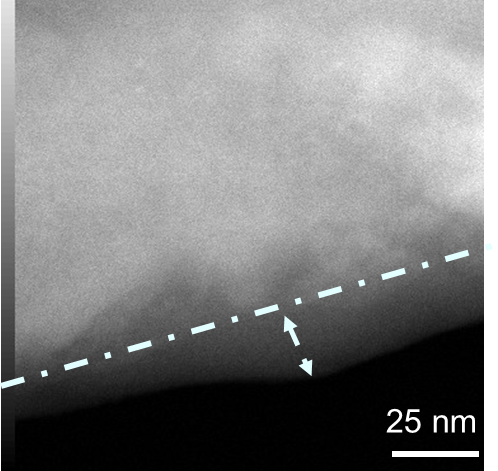


**Fig. S12** Dark-field STEM image of the Si anode cycled in P electrolyte for the EDS mapping in the Fig. 3d


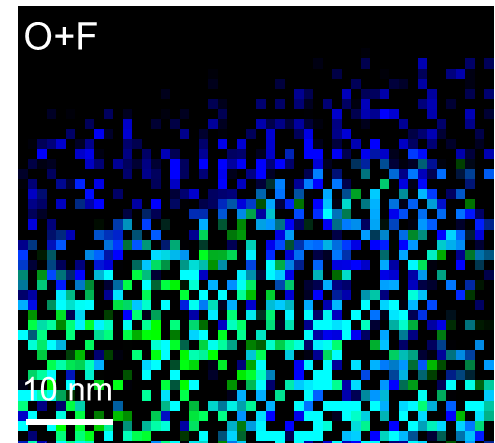


**Fig. S13** The distribution of F and O based on the EELS mapping of the Si particle cycled in PF electrolyte


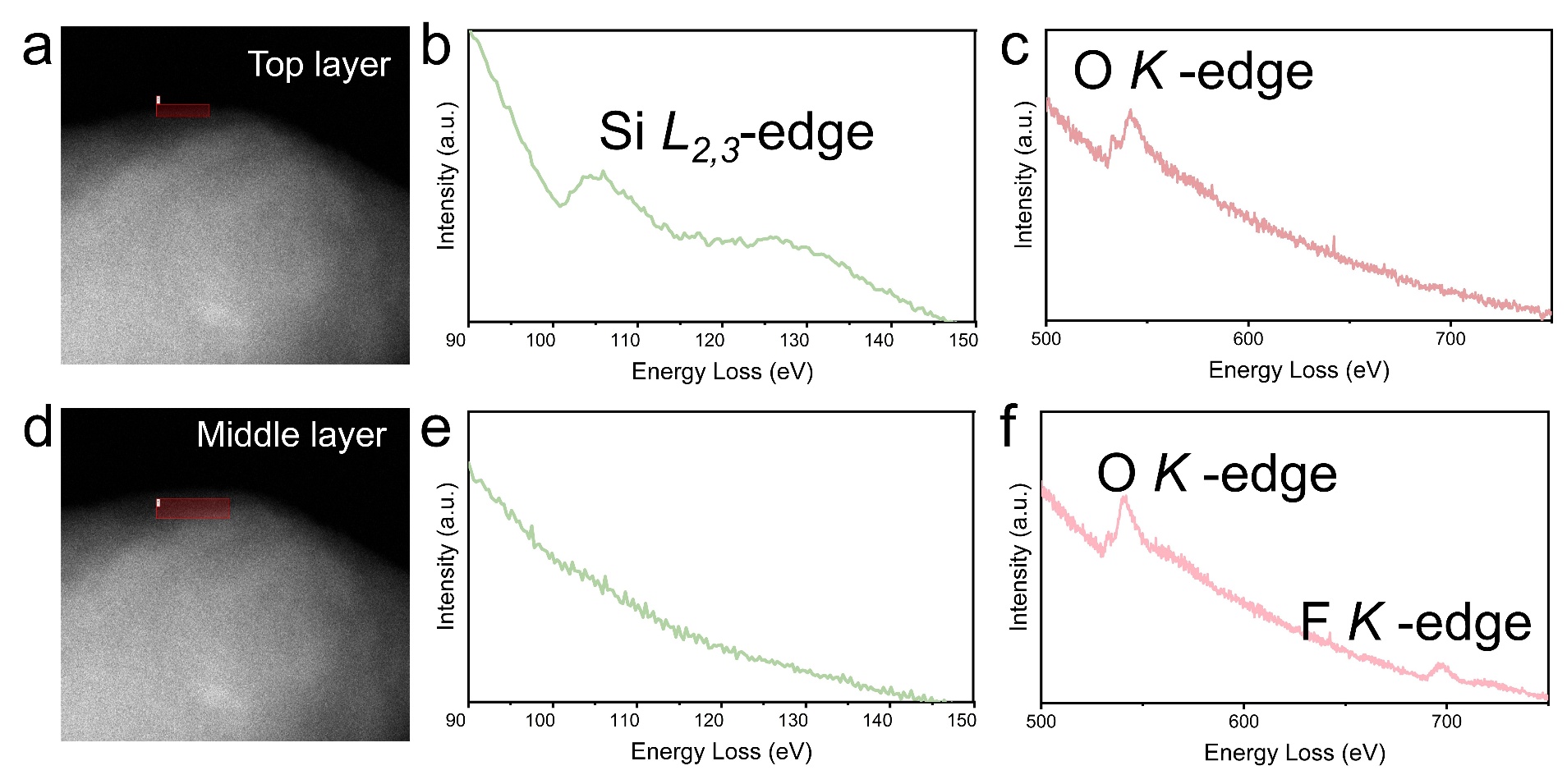


**Fig. S14** EELS spectra from the top layer (**a-c**) and middle layer (**d-f**) of the Si particle in the PF electrolyte


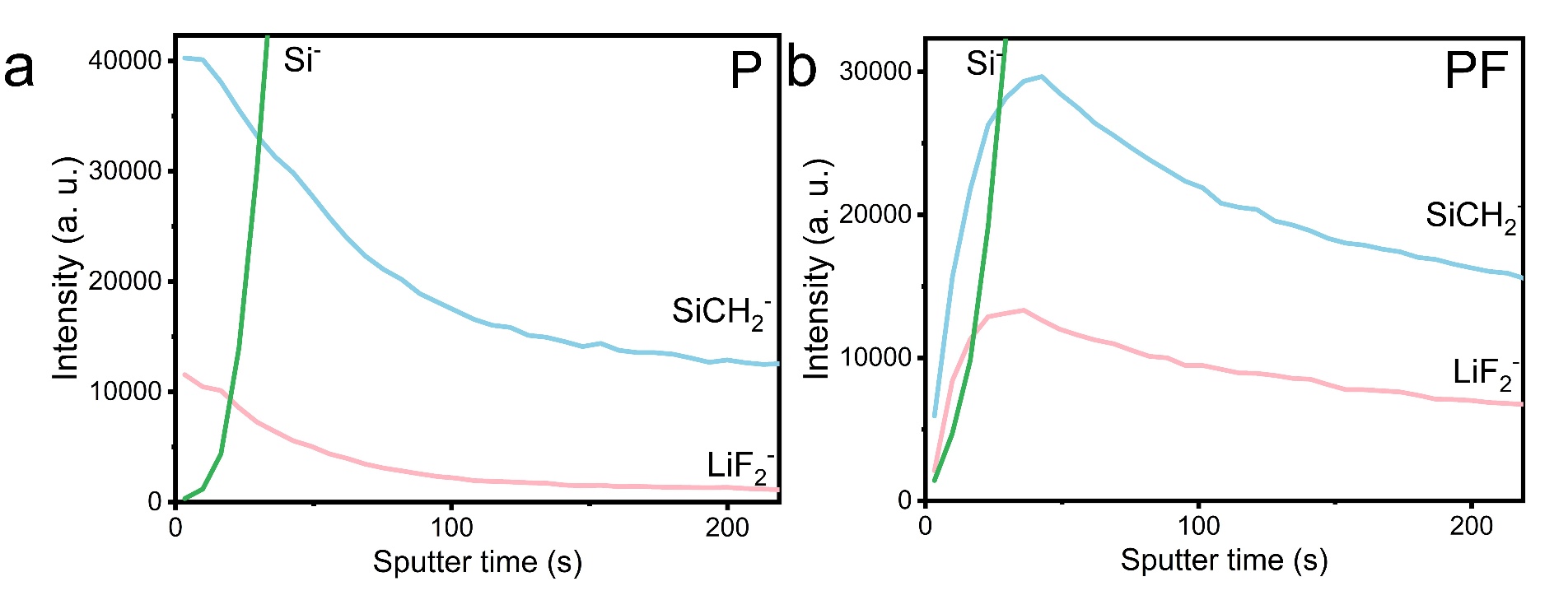


**Fig. S15** ToF-SIMS spectra of the Si^–^, LiF₂^–^, and SiCH₂^–^signals of P electrolyte (**a**) and PF electrolyte (**b**)


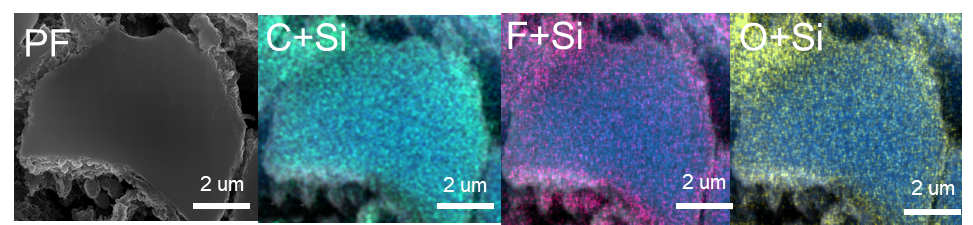


**Fig. S16.** Cross-sectional image and its corresponding elemental mappings based on the EDS from the Si anode after 50 cycles in the PF electrolyte


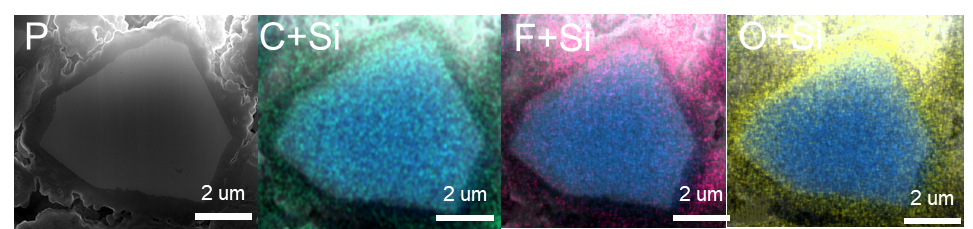


**Fig. S17** Cross-sectional image and its corresponding elemental mappings based on the EDS from the Si anode after 50 cycles in the P electrolyte


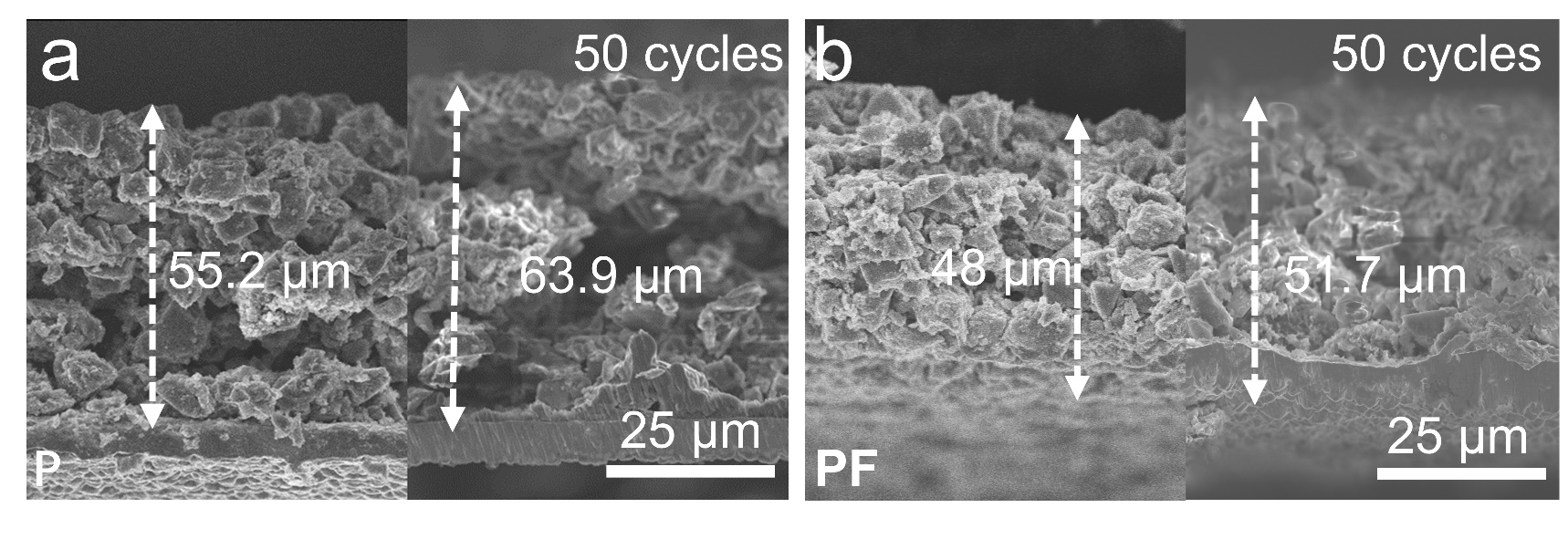


**Fig. S18** Cross-sectional SEM images of the Si anodes cycled in the (**a**) P and (**b**) PF electrolytes after 3 cycles at 50 mA g^–1^ and 50 cycles at 200 mA g^–1^


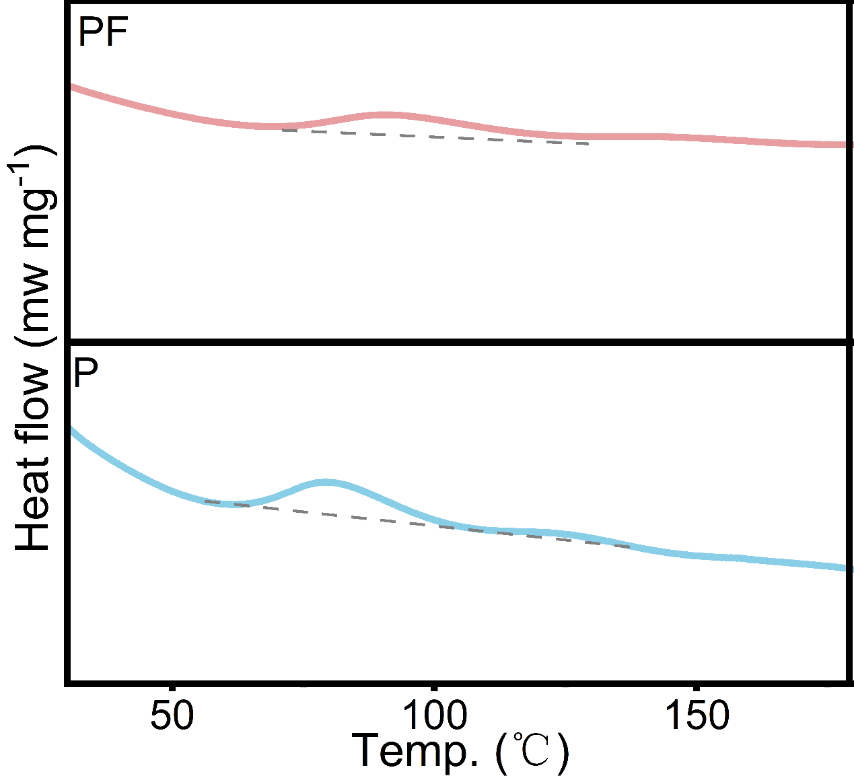


**Fig. S19** DSC curves of the Si electrodes after 10 cycles in the P and PF electrolytes


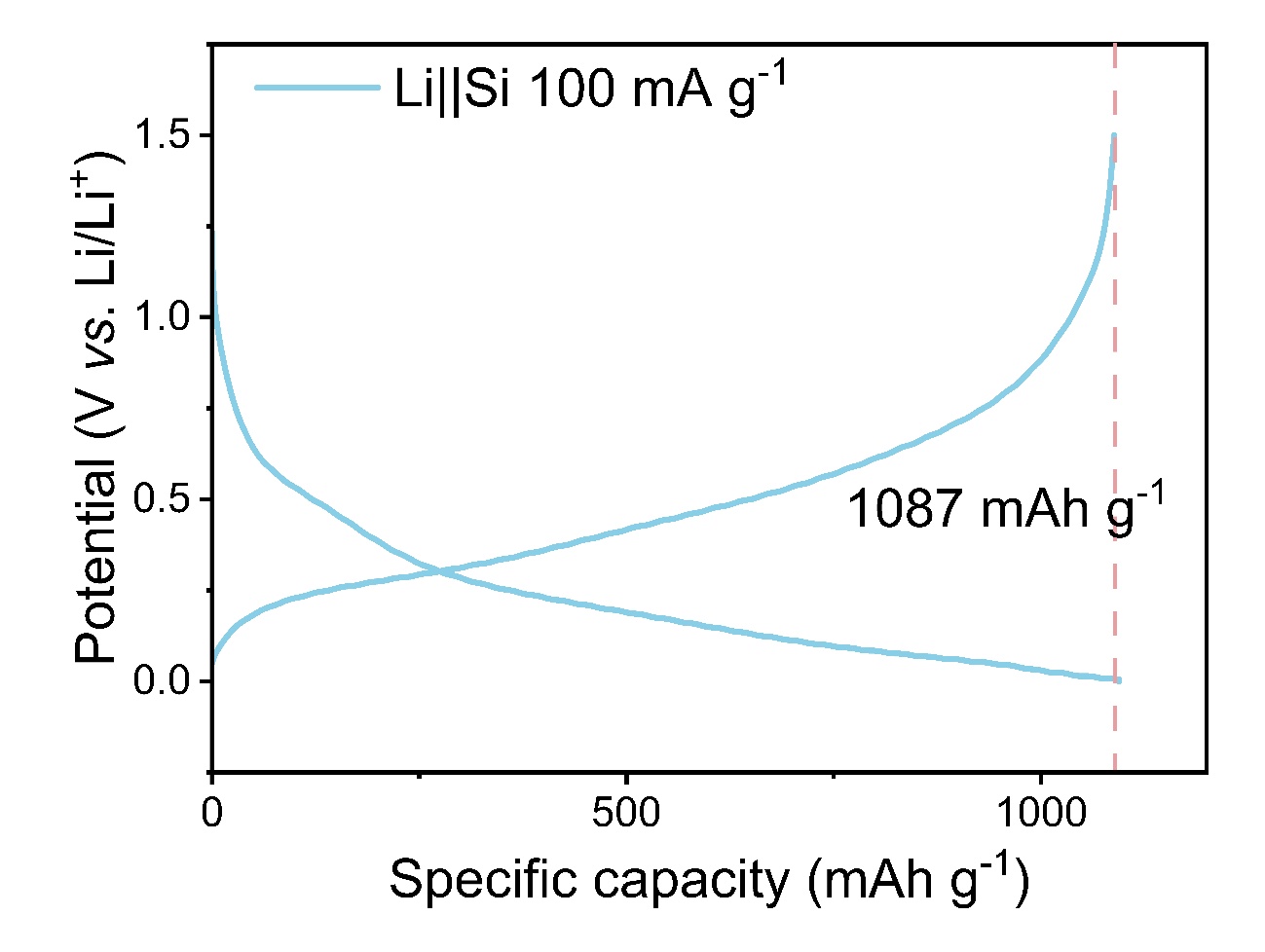


**Fig. S20** Galvanostatic charge-discharge profiles of Li||Si half-cells at a current density of 100 mA g⁻¹ in the voltage range of 0.005–1.5 V


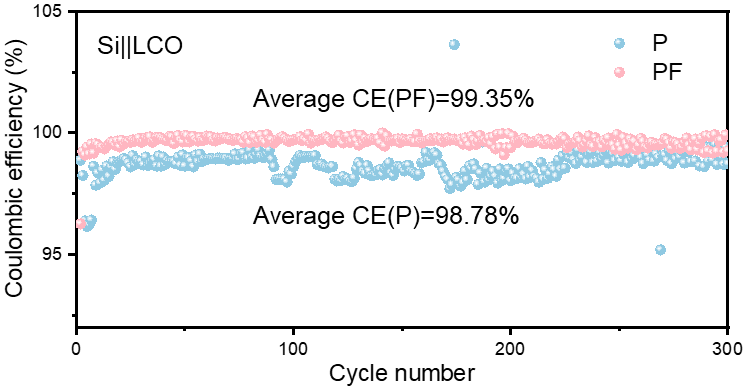


**Fig. S21** the CE changes as a function of cycle number of the Si||LCO full cells with the P and PF electrolyte at 100 mA g^–1^


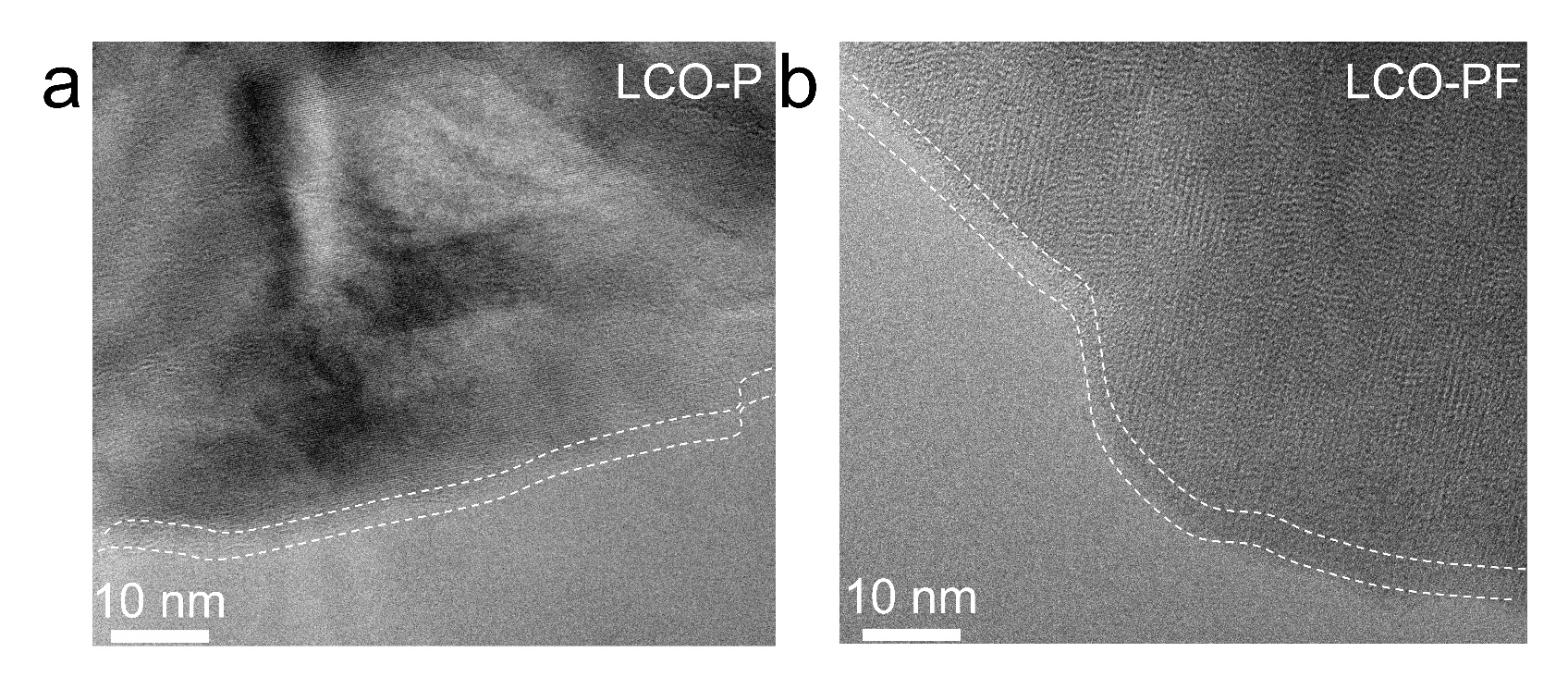


**Fig. S22** Cryo-TEM images of LCO cathodes cycled for 100 cycles in Si ∥ LCO full cells with (a) P electrolyte and (b) PF electrolyte after 10 cycles


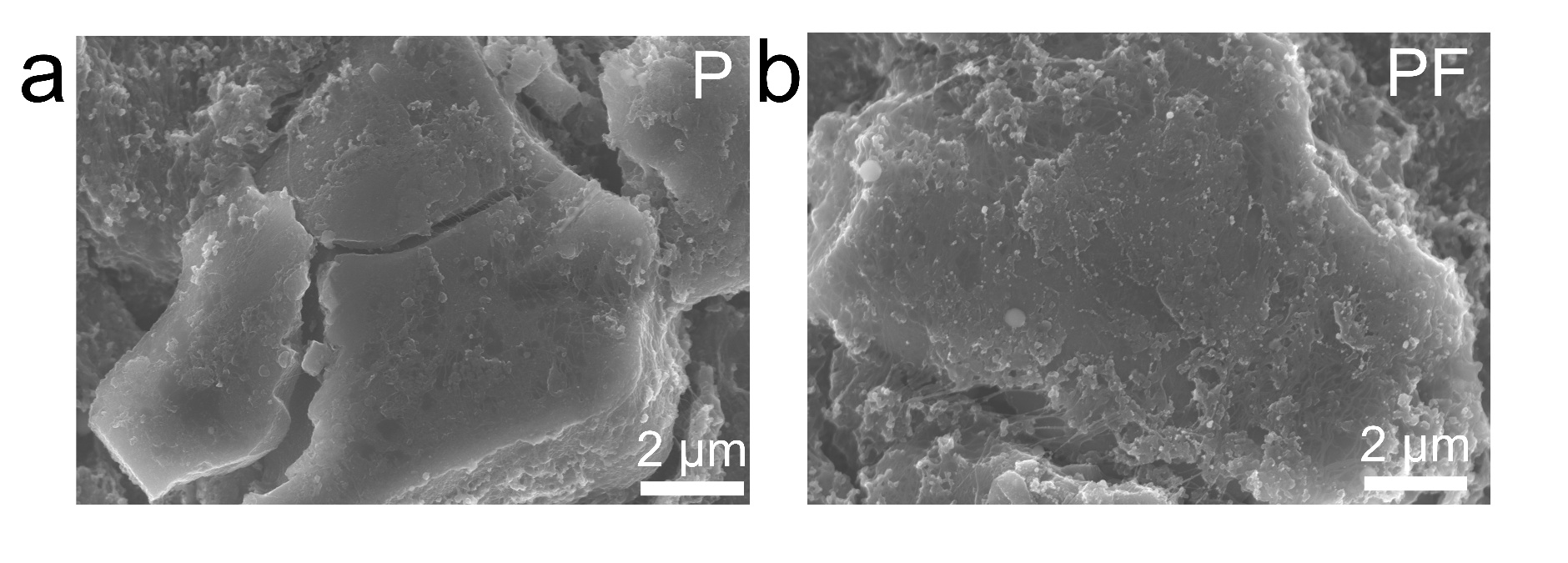


**Fig. S23** Top-view SEM images of Si anodes after 300 cycles at 100 mA g⁻¹ in Si||LiCO full cell in P electrolyte (a) and PF electrolyte (b)


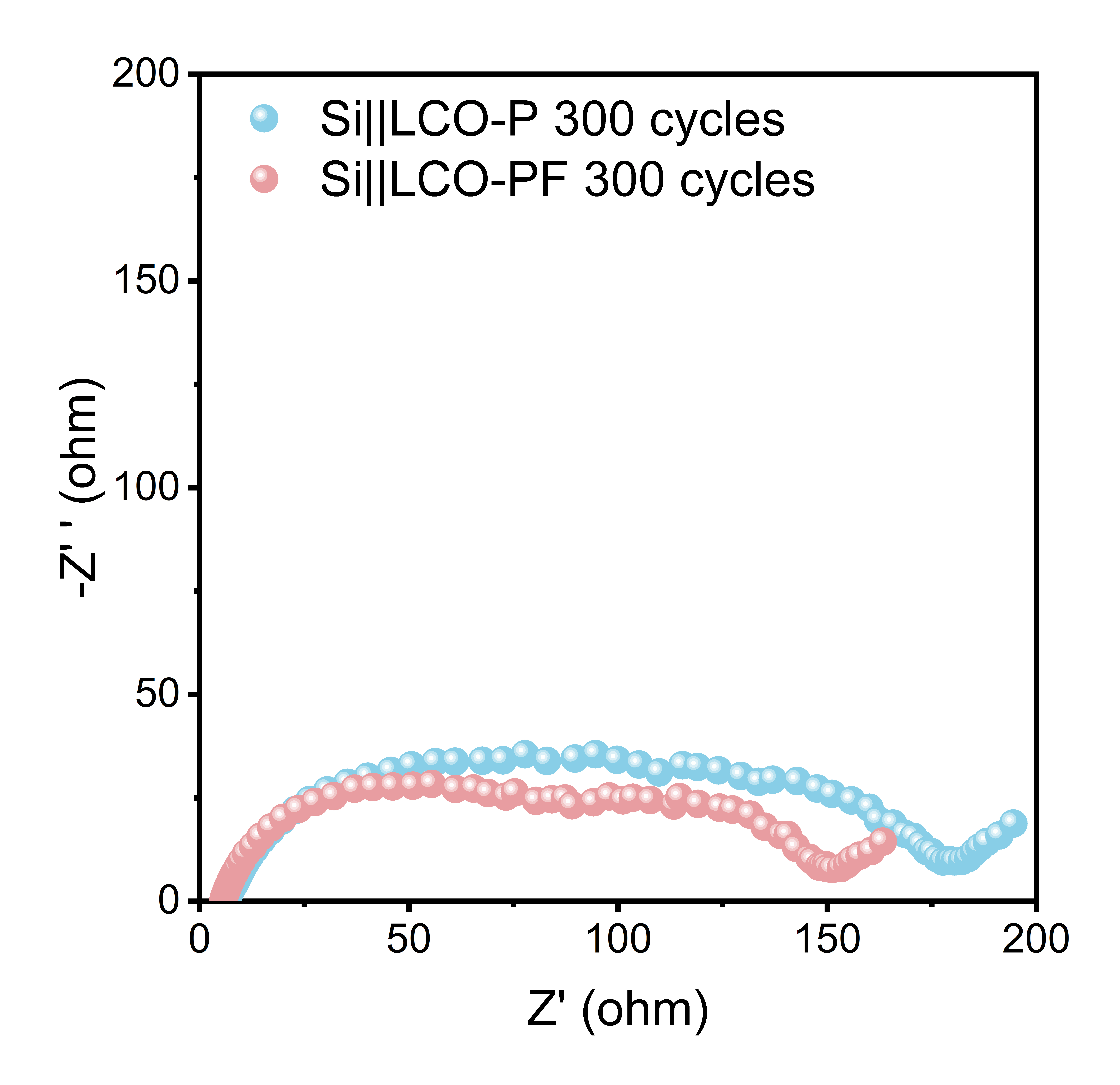


**Fig. S24** EIS measurements on the Si||LCO full cell after 300 cycles


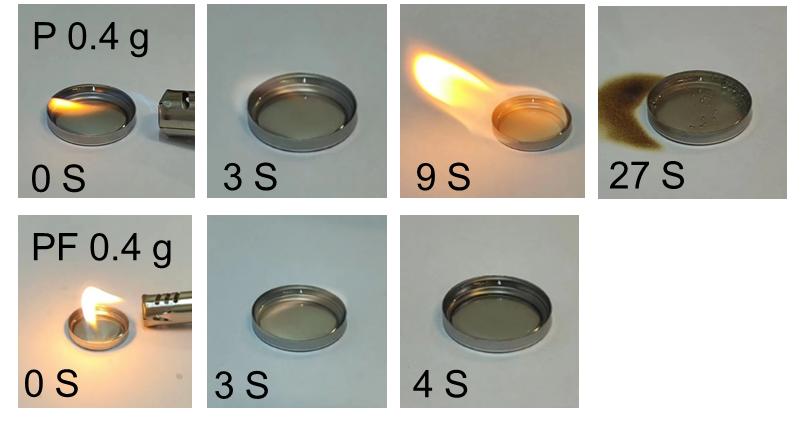


**Fig. S25** Flammability tests for the P and PF electrolyte


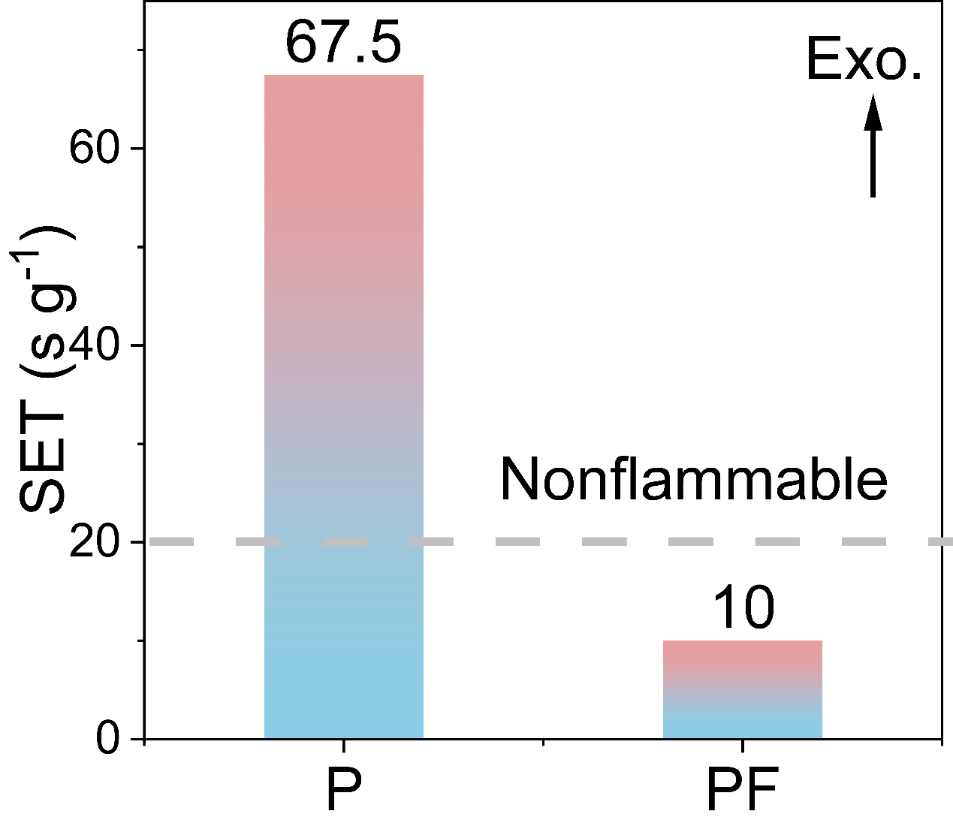


**Fig. S26** SET value for the P and the PF electrolyte


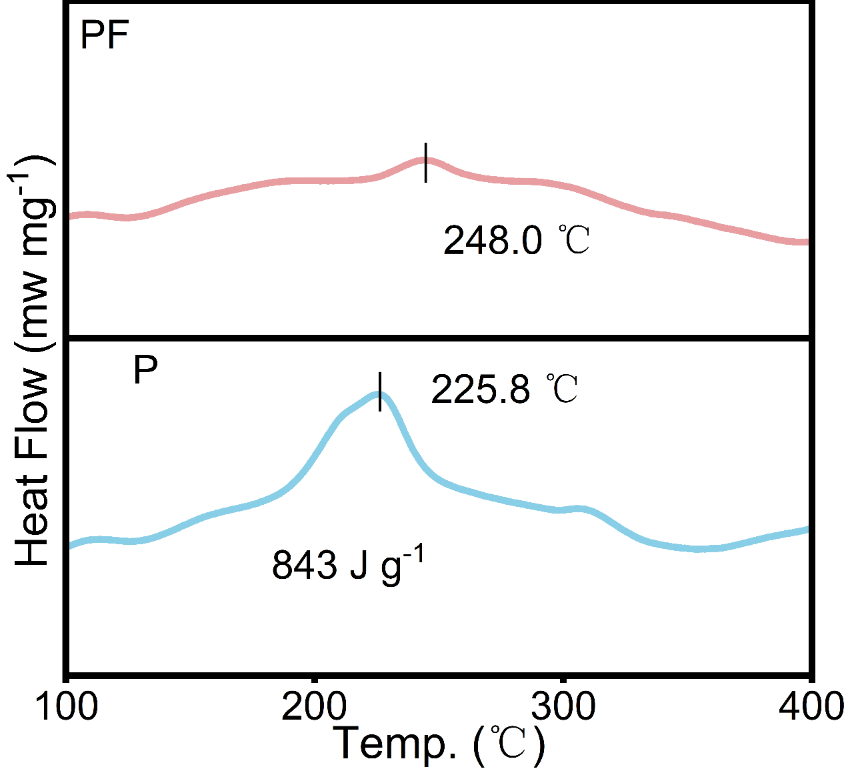


**Fig. S27** DSC curves of the P and PF electrolyte


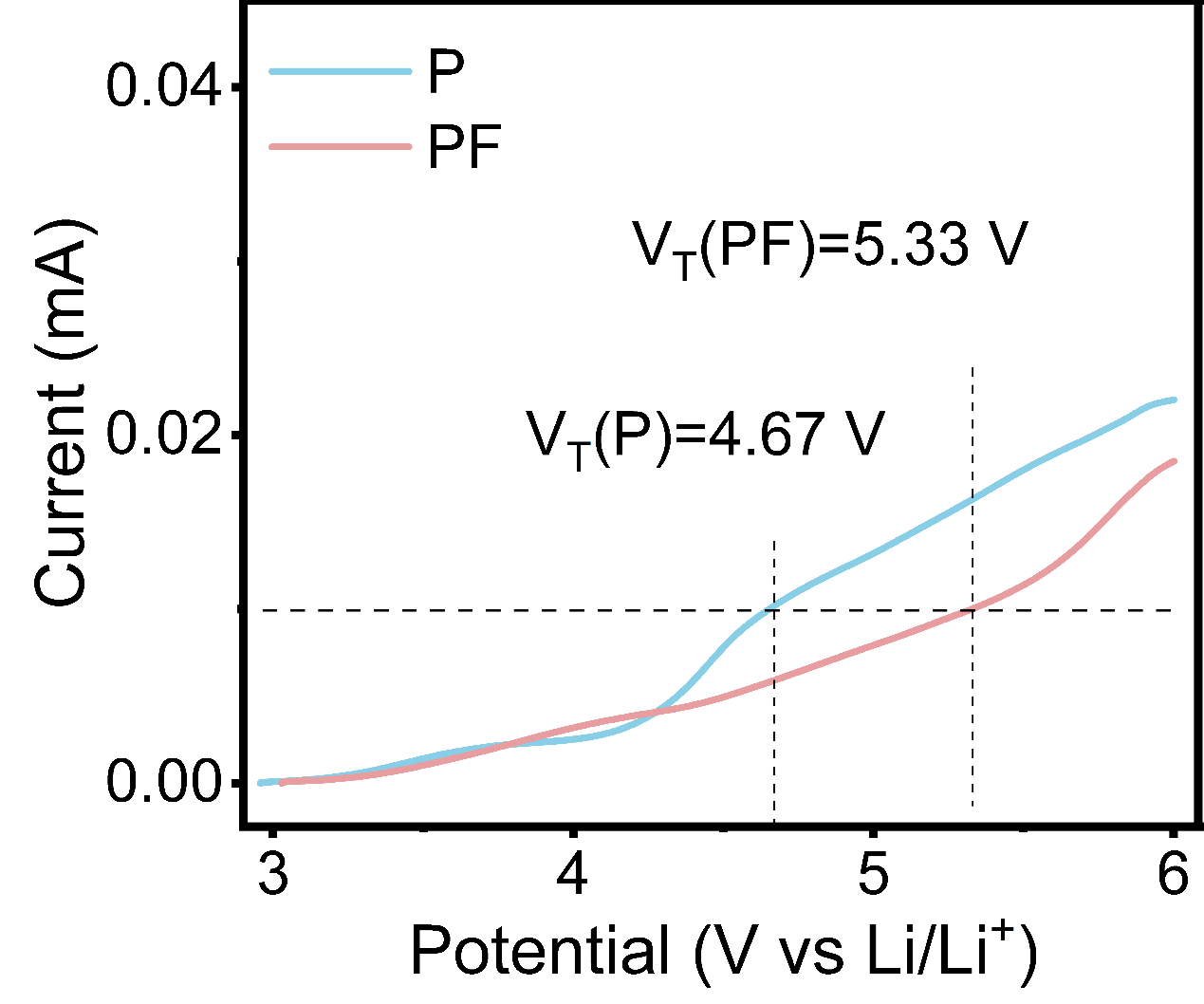


**Fig. S28** LSV curves for the P and PF electrolyte and their V_T_

**Table S1** Equivalent circuits and EIS fitting results and errors





| **Sample** | ***R_s_*** | ***Error%*** | ***R_SEI/CEI_*** | ***Error%*** | ***R_ct_*** | ***Error%*** |
| --- | --- | --- | --- | --- | --- | --- |
| **Li\|\|Si 10^th^ cycles-P** | 3.2 | 0.7 | 3.4 | 1.0 | 5.9 | 0.3 |
| **Li\|\|Si 10th cycles-PF** | 3.3 | 0.6 | 2.4 | 1.6 | 4.7 | 0.5 |
| **Si\|\|LCO 10^th^ cycles-P** | 4.0 | 0.6 | 10.3 | 0.5 | 44.45 | 1.4 |
| **Si\|\|LCO 10^th^ cycles-PF** | 4.1 | 0.5 | 13.6 | 1.2 | 20 | 1.5 |
| **Si\|\|LCO 300^th^ cycles-P** | 7.6 | 0.3 | 57.6 | 6.0 | 104.7 | 4.3 |
| **Si\|\|LCO 300^th^ cycles-PF** | 6.9 | 0.5 | 52.46 | 4.1 | 78 | 3.6 |

**Table S2** Performance comparison of the stabilization strategies for silicon (Si) anodes between this work and other reported studies

| Materials | Strategy | Current | cycles | Capacity retention | Refs. |
| --- | --- | --- | --- | --- | --- |
| Si | **LiF-Pie SEI** | **0.5 C** | **300** | **88.9%** | **This work** |
| Si-SiO_x_/C | CNT nanoskeleton | 0.5 C | 300 | 72% | [S1] |
| Nano Si | Si@TiO_2_ | 0.5 C | 100 | 53% | [S2] |
| Si-C | Multi-wall CNTs coating | 0.5 C | 100 | 76% | [S3] |
| Si | Pre-lithiation | 1 C | 300 | 82% | [S4] |
| SiOx-C | 3D porous SiOx/C composites | 0.5 C | 100 | 88.9% | [S5] |
| Si | Fe crosstalk | 0.33 C | 100 | 52% | [S6] |
| Si | Polyaniline coating | 0.2 C | 45 | 80% | [S7] |
| Micro-Si | Selective dissolution of SEI components | 0.05 C | 150 | 83.7% | [S8] |
| Si | silver -decorated mucic acid buffer interface | 0.2 C | 100 | 87% | [S9] |
| Si | trifunctional network binder | 0.2 C | 140 | 57% | [S10] |

**Supplementary References**

1. X. Guan, Y. Zhang, I.A. Kinloch, M.A. Bissett, “Nanoskeleton” Si-SiO*_x_*/C anodes toward highly stable lithium-ion batteries. ACS Appl. Mater. Interfaces **17**(7), 10580–10592 (2025). <https://doi.org/10.1021/acsami.4c18254>
2. Y. Jin, S. Li, A. Kushima, X. Zheng, Y. Sun et al., Self-healing SEI enables full-cell cycling of a silicon-majority anode with a coulombic efficiency exceeding 99.9%. Energy Environ. Sci. **10**(2), 580–592 (2017). <https://doi.org/10.1039/c6ee02685k>
3. M. Choi, J. Sung, G. Yeo, S. Chae, M. Ko, A strategy of boosting the effect of carbon nanotubes in graphite-blended Si electrodes for high-energy lithium-ion batteries. J. Energy Storage **72**, 108301 (2023). <https://doi.org/10.1016/j.est.2023.108301>
4. P. Qiu, M. Cui, H. Gan, L. Li, Y. Xia et al., Hierarchically structured silicon–carbon anodes: Achieving high-performance all-solid-state Li-ion batteries *via* chemical pre-lithiation and *in situ* polymerization. Carbon **233**, 119905 (2025). <https://doi.org/10.1016/j.carbon.2024.119905>
5. S. Kuang, D. Xu, W. Chen, X. Huang, L. Sun et al., *In situ* construction of bamboo charcoal derived SiOx embedded in hierarchical porous carbon framework as stable anode material for superior lithium storage. Appl. Surf. Sci. **521**, 146497 (2020). <https://doi.org/10.1016/j.apsusc.2020.146497>
6. M. Kim, S.P. Harvey, Z. Huey, S.-D. Han, C.-S. Jiang et al., A new mechanism of stabilizing SEI of Si anode driven by crosstalk behavior and its potential for developing high performance Si-based batteries. Energy Storage Mater. **55**, 436–444 (2023). <https://doi.org/10.1016/j.ensm.2022.12.004>
7. S. Pan, J. Han, Y. Wang, Z. Li, F. Chen et al., Integrating SEI into layered conductive polymer coatings for ultrastable silicon anodes. Adv. Mater. **34**(31), 2203617 (2022). <https://doi.org/10.1002/adma.202203617>
8. Y.-F. Tian, S.-J. Tan, C. Yang, Y.-M. Zhao, D.-X. Xu et al., Tailoring chemical composition of solid electrolyte interphase by selective dissolution for long-life micron-sized silicon anode. Nat. Commun. **14**(1), 7247 (2023). <https://doi.org/10.1038/s41467-023-43093-6>
9. L. Lv, Y. Wang, W. Huang, Y. Li, Q. Shi et al., Construction of a LiF-rich and stable SEI film by designing a binary, ion-, and electron-conducting buffer interface on the Si surface. ACS Appl. Mater. Interfaces **14**(30), 35246–35254 (2022). <https://doi.org/10.1021/acsami.2c08019>
10. Z. Li, Y. Zhang, T. Liu, X. Gao, S. Li et al., Silicon anode with high initial coulombic efficiency by modulated trifunctional binder for high-areal-capacity lithium-ion batteries. Adv. Energy Mater. **10**(20), 1903110 (2020). <https://doi.org/10.1002/aenm.201903110>
